# Metagenomics of Thermophiles with a Focus on Discovery of Novel Thermozymes

**DOI:** 10.3389/fmicb.2016.01521

**Published:** 2016-09-27

**Authors:** María-Eugenia DeCastro, Esther Rodríguez-Belmonte, María-Isabel González-Siso

**Affiliations:** Grupo EXPRELA, Centro de Investigacións Científicas Avanzadas (CICA), Departamento de Bioloxía Celular e Molecular, Facultade de Ciencias, Universidade da CoruñaA Coruña, Spain

**Keywords:** metagenomics, thermophiles, thermozymes, bioinformatics, NGS

## Abstract

Microbial populations living in environments with temperatures above 50°C (thermophiles) have been widely studied, increasing our knowledge in the composition and function of these ecological communities. Since these populations express a broad number of heat-resistant enzymes (thermozymes), they also represent an important source for novel biocatalysts that can be potentially used in industrial processes. The integrated study of the whole-community DNA from an environment, known as metagenomics, coupled with the development of next generation sequencing (NGS) technologies, has allowed the generation of large amounts of data from thermophiles. In this review, we summarize the main approaches commonly utilized for assessing the taxonomic and functional diversity of thermophiles through metagenomics, including several bioinformatics tools and some metagenome-derived methods to isolate their thermozymes.

## Introduction

Thermophiles (growing optimally at 50°C or higher), extreme thermophiles (65–79°C) and hyperthermophiles (above 80°C), categories defined per Wagner and Wiegel (2008), are naturally found in various geothermally heated regions of Earth such as hot springs and deep-sea hydrothermal vents. They can also be present in decaying organic matter like compost and in some man-made environments. Besides the high temperatures, many of these environments are characterized by extreme pH or anoxia. The adaptation to these harsh habitats explains the high genomic and metabolic flexibility of microbial communities in these ecosystems (Badhai et al., 2015) and makes thermophiles and their thermostable proteins very suitable for some industrial and biotechnological applications. Therefore, screening for novel biocatalysts from extremophiles has become a very important field. In the last few years, novel thermostable polymerases (Moser et al., 2012; Schoenfeld et al., 2013), beta-galactosidases (Wang et al., 2014), esterases (Fuciños et al., 2014), and xylanases (Shi et al., 2013), among others, have been described and characterized, opening a new horizon in biotechnology.

Apart from the bioprospecting purposes, the analysis of these high-temperature ecosystems and their inhabitants can improve our understanding of microbial diversity from an ecological point of view and increase our knowledge of heat-tolerance adaptation (Lewin et al., 2013). Additionally, the study of thermophiles provides a better comprehension about the origin and evolution of earliest life, as they are considered to be phenotypically most similar to microorganisms present on the primitive Earth (Farmer, 1998; Stetter, 2006). In addition to the bacterial and archaeal communities, there is an increasing interest in the study of the viral populations living in high-temperature ecosystems, as viruses are reported to be the main predators of prokaryotes in such environments (Breitbart et al., 2004), participating in the biogeochemical cycles and being important exchangers of genetic information (Rohwer et al., 2009).

The first studies of these extremophiles required their cultivation and isolation (Morrison and Tanner, 1922; Brock and Freeze, 1969; Fiala and Stetter, 1986; Prokofeva et al., 2005; De la Torre et al., 2008). Although these techniques have been improved (Tsudome et al., 2009; Pham and Kim, 2012), the growth of thermophiles under laboratory conditions is still a limitation for the insights into the microbial diversity. The evolution of high-throughput DNA sequencing has enabled the development and improvement of metagenomics: the genomic analysis of a population of microorganisms (Handelsman, 2004). Different high-temperature ecosystems like hot springs (Schoenfeld et al., 2008; Gupta et al., 2012; Ghelani et al., 2015; López-López et al., 2015b; Sangwan et al., 2015), deserts (Neveu et al., 2011; Fancello et al., 2012; Adriaenssens et al., 2015), compost (Martins et al., 2013; Verma et al., 2013), hydrocarbon reservoirs (de Vasconcellos et al., 2010; Kotlar et al., 2011), hydrothermal vents (Anderson et al., 2011, 2014), or a biogas plant (Ilmberger et al., 2012) have been analyzed using this metagenomic approach. These whole community DNA based studies were initially focused to answering the question “who are there” and now have shifted to finding out “what are they doing,” allowing us the access to the natural microbial communities and their metabolic potential (Kumar et al., 2015).

## Diversity analysis of thermophiles

### Targeted metagenomics

The universality of the 16S rRNA genes makes them an ideal target for phylogenetic analysis and taxonomic classification (Olsen et al., 1986). Schmidt et al. (1991) were the pioneers in performing a community characterization based on metagenome amplified 16S rRNA genes. Since then, the diversity of other natural microbial communities started to be studied using this approach. Jim's Black Pool hot spring, in Yellowstone National Park (YNP), is reported to be the first metagenome-derived analysis of a high-temperature environment based on 16S rRNA gene profiling (Barns et al., 1994).

**Figure 1:**
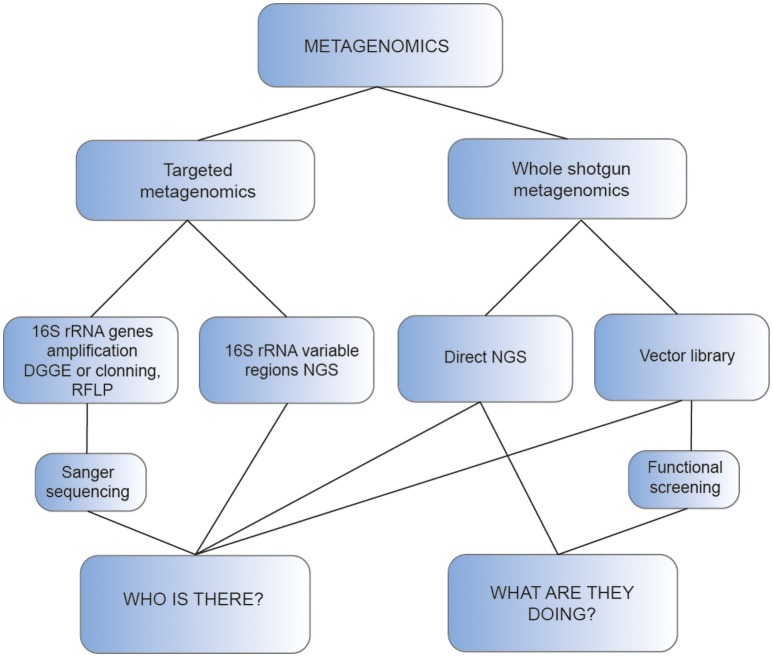
**Schematic representation of the main approaches used for metagenomic analysis of thermophiles**.

Initially, these studies required the amplification of the 16S rRNA genes followed by either denaturing gradient gel electrophoresis (DGGE, Muyzer et al., 1993) and sequencing or by cloning of the amplicons. In this case, the libraries obtained were screened using direct Sanger sequencing or restriction fragment length polymorphism (RFLP) analysis (Liu et al., 1997; Baker et al., 2001), to select and sequence those clones with unique patterns (Figure 1). As an example, the effect of pH, temperature, and sulfide in the hyperthermophilic microbial communities living in hot springs of northern Thailand was determined with the amplification of complete 16S rRNA genes followed by DGGE separation and sequencing (Purcell et al., 2007). In a different study, RFLP analysis and sequencing of clones with unique RFLP patterns was used to reveal the presence of abundant novel *Bacteria* and *Archaea* sequences in a 16S rRNA gene clone library prepared from the 55°C water and sediments of Boiling Spring Lake in California, USA (Wilson et al., 2008).

With the development of next generation sequencing (NGS) technologies, more samples can be analyzed at lower sequencing cost and time, improving the production of 16S rRNA gene-based biodiversity studies. Additionally, the use of NGS allows to recover more information about the taxonomy of the sample, as reflected by Song et al. (2013), who obtained greater detail in the community structures from 16 Yunnan and Tibetan hot springs with high throughput 454-pyrosequencing than previous studies using conventional clone library and DGGE (Song et al., 2010). These analyses often rely on a partial sequence of 16S rRNA genes, as the read length of most NGS platforms is relatively short. For this purpose, primers designed for amplification of variable regions of 16S rRNA, like the V4–V8 (Hedlund et al., 2013; Huang et al., 2013), or the V3–V4 (Chan et al., 2015) are used. In the last few years, a high amount of extreme temperature environments have been analyzed with this procedure, especially hot springs, some of which are summarized in Table 1. Thanks to this strategy, a large number of 16S rRNA sequences have been produced and deposited in public databases like the Ribosomal Database Project (RDP, Cole et al., 2014) or the SILVA database (Quast et al., 2013).

**Table 1:** **Examples of hot springs studied using the amplification of the variable regions of 16S rRNA**.

**Hot spring**	**Type of sample**	**pH**	**Temperature (°C)**	**Sequencer**	**Region amplified**	**References**
Siloam, Limpopo, South Africa	Water	9.5	63	Roche 454 GS FLX	V4–V7	Tekere et al., 2011
Lake Bogoria, Kenya	Water, sediment and microbial mat	8.9–9.5	40–80	Roche 454 GS FLX	V3–V4	Dadheech et al., 2013
Arzakan and Jermuk, Armenia	Water and sediment	7.20–7.50	40–53	Roche 454 GS FLX	V4–V8	Hedlund et al., 2013
Bacon Manito Geothermal Field, Philippines	Sediment	3.72–6.58	60–92	Roche 454 GS FLX	V4–V8	Huang et al., 2013
Furnas Valley, Saõ Miguel, Azores	Water, sediment and microbial mat	2.5–8	51–92	Roche 454 GS FLX	V2–V3	Sahm et al., 2013
Yunnan province and Tibet, China	Sediment	3.2–8.6	47–96	Roche 454 GS FLX	V4	Song et al., 2013
Zavarzin, Uzon Caldera, Kamchatka, Russia	Microbial mat	6.6	56–58	Roche 454 GS FLX	V3	Rozanov et al., 2014
Sungai Klah, Malaysia	Water and sediment	8	75–85	Illumina MiSeq	V3–V4	Chan et al., 2015
Jakrem, Meghalaya, India	Microbial mat	–	–	Illumina	V3	Panda et al., 2015
Odisha, Deulajhari, India	Sediment	7.14–7.83	43–55	Illumina GAIIX	V3–V4	Singh and Subudhi, 2016

Even when the process of generating and sequencing the libraries is relatively fast, this PCR-based approach is biased due to limitations of primers, PCR artifacts like chimeras (Ashelford et al., 2005) and inhibitors that could be present in the sample hindering the amplification (Urbieta et al., 2015). Although there are some previous studies focused on primer design to acquire a high coverage rate (Wang and Qian, 2009), difficulties of the primers in recognizing all the 16S rRNA sequences have been described (Cai et al., 2013), leading to the unequal amplification of species 16S rRNA genes. Furthermore, analysis of 16S rRNA sequences can result in misidentification of the taxonomy, as closely related species may harbor nearly identical 16S rRNA genes. In addition, an overestimation of the community diversity could occur since sporadic cases of distant horizontal transfer of the 16S rRNA gene have been inferred from comparisons of these genes within and between individual genomes (Yap et al., 1999; Acinas et al., 2004).

The most used taxonomically informative genomic marker in targeted metagenomics is 16S rRNA, but there are other signature sequences that have been used to study the diversity of thermophiles such as internal transcribed spacer regions (ITS, Ferris et al., 2003) or 18S rRNA genes (Wilson et al., 2008), as well as different protein-coding genes such as *aox*B gene fragment, which encodes the catalytic subunit of As(III) oxidase, employed by Sharma et al. (2015) in combination to 16S rRNA to assess the microbial diversity of the Soldhar hot spring in India.

Apart from the above mentioned amplicon-targeting strategy, in some studies a sequence capture technique coupled with NGS is driven to enrich the targeted sequences present in the metagenome. Captured metagenomics involves custom-designed hybridization-based oligonucleotide probes that hybridize with the metagenomic libraries followed by the sequencing of the probe-bound DNA fragments. Denonfoux et al. (2013) firstly used this procedure to explore the methanogen diversity in Lake Pavin (Frech Massif Central), showing that this GC-independent procedure is less biased and can detect broader diversity than traditional amplicon sequencing. The same approach has been used to enhance the capture of functional genes coding for carbohydrate-active enzymes and proteases in agricultural soils (Manoharan et al., 2015), and could also be an interesting tool to study thermophilic populations.

Another method for targeted metagenomics enrichment is stable isotope probing (SIP) in which the environmental microorganisms are grown in the presence of substrates labeled with isotopes. As a consequence of metabolic activity, the isotope (usually ^13^C or ^15^N) is incorporated into the nucleic acids of the microbes metabolizing the substrate, increasing the density of DNA or RNA that can be after separated from unlabelled ones (Coyotzi et al., 2016). The high-density community DNA is then used as template to amplify by PCR the 16S rRNA sequences (Brady et al., 2015) and/or some functional genes involved in the selected metabolic pathway, thus allowing the study of the microorganisms that are actively participating in the processes of interest. Gerbl et al. (2014) used this technique to assess the microbial populations implicated in the carbon cycle in the Franz Josef Quelle radioactive thermal spring (Austrian Central Alps).

Although the strategies of targeted metagenomics can be used to infer the taxonomic diversity of the community (16S rRNA gene profiling) or particular aspects of its functional diversity, a broader view of functional diversity, i.e., a more exhaustive answer to the question “what are they doing,” is provided by shotgun metagenomics (Figure 1).

### Shotgun metagenomics

Random sequencing of metagenomic DNA using high-throughput sequencing technology is becoming increasingly common. In this approach, DNA is extracted from the whole community and subsequently sheared into small fragments that are independently sequenced. At present, this is considered the most accurate method for assessing the structure of an environmental microbial community, since it does not comprise any selection and reduces technical biases, especially the ones introduced by amplification of the 16S rRNA gene (Lewin et al., 2013). Shah et al. (2011) compared bacterial communities analyzed with both 16S rRNA and whole shotgun metagenomics, revealing that the taxonomy derived from these two different approaches cannot be directly compared. This study also proposed that low abundance species are best identified through 16S rRNA gene sequencing. Therefore, some high-temperature studies use, in parallel, both techniques to assess the taxonomic composition of the microbial community (Dadheech et al., 2013; Klatt et al., 2013; Chan et al., 2015).

The biodiversity of several hot environments such as oil reservoirs (Kotlar et al., 2011), compost (Martins et al., 2013), or hot springs (Zamora et al., 2015; Mehetre et al., 2016), was studied using shotgun metagenomics sequencing. Some of them are summarized in Table 2.

**Table 2:** **Examples of high temperature environments studied with shotgun metagenomics**.

**Environment**	**Location**	**Type of sample/s**	**pH**	**Temperature (°C)**	**Sequencer**	**Total reads**	**Size (Mbp)**	**References**
Hot spring	Yellowstone National Park, USA	Microbial mat	–	60–65	Sanger	161,976	167	Klatt et al., 2011
	Yellowstone National Park, USA	Microbial mat	6.2–9.1	40–60	Sanger	–	320.6	Klatt et al., 2013
	Yellowstone National Park, USA	Microbial mat	3.5	60–78	Roche 454	–	–	Kozubal et al., 2013
	Yellowstone National Park, USA	Microbial mat	2.5–7.8	65–80	Sanger	75,000	60	Inskeep et al., 2010
	Yellowstone National Park, USA	Microbial mat and sediment	2.5–6.4	70–85	Sanger	–	250	Inskeep et al., 2013
	Yellowstone National Park, USA	Water	1.8	79	Roche 454	1,604,079	–	Menzel et al., 2015
	Yellowstone National Park, USA	Water	3.5–4.0	92	Roche 454	420,726	–	Menzel et al., 2015
	Yellowstone National Park, USA	Microbial mat	7.9	80–82	Sanger	–	1.29	Colman et al., 2016
	Los Azufres, Mexico	Sediment	3.6	75	Illumina GaIIx	6,000,792	216	Servín-Garcidueñas et al., 2013
	Lake Bogoria, Kenya	Water, sediment and microbial mat	8.9–9.5	40–80	Roche 454	24,567	12.7	Dadheech et al., 2013
	Saõ Miguel, Azores	Water, sediment and microbial mat	2.5–8	51–92	Roche 454	–	–	Sahm et al., 2013
	Champagne pool, New Zealand	Water and sediment	5.5–6.9	45–75	Illumina MiSeq	4,623,251	–	Hug et al., 2014
	Long Valley Caldera, California	Microbial mat	–	50–80	Illumina MiSeq	–	–	Stamps et al., 2014
	Odisha, India	Water and sediment	7.2–7.4	40–58	Roche 454 GS	–	71.26	Badhai et al., 2015
	Sungai Klah, Malaysia	Water and sediment	8.00	75–85	Illumina HiSeq	5,527,175,000	–	Chan et al., 2015
	Shi-Huang-Ping, Taiwan	Water	2.5	69	Illumina HiSeq	557,415,266	–	Lin et al., 2015
	Lobios, Ourense, Spain	Water	8.2	76	Illumina HiSeq	11,982,436	–	López-López et al., 2015b
	Tuwa, India	Water	8.2–9	54–65	Ion Torrent PGM	541,379	98.7	Mangrola et al., 2015a
	Lasundra, India	Water	6.0	42–52	Ion Torrent PGM	606,867	98.6	Mangrola et al., 2015b
	Eryuan, China	Sediment	7.0	65	Illumina HiSeq	10,360,000	–	Menzel et al., 2015
	Uzon Caldera, Russia	Water and sediment	5.8–6.0	61–64	Roche 454	660,054	–	Menzel et al., 2015
	Pozzuoli, Italy	Water and sediment	3.0	76	Illumina HiSeq	10,060,000	–	Menzel et al., 2015
	Pisciarelly, Italy	Sediment	5.5	86	Roche 454	876,681	–	Menzel et al., 2015
	Grensdalur, Iceland	Water and sediment	5.0	85–90	Illumina HiSeq	10,330,000	–	Menzel et al., 2015
	Krísuvík, Iceland	Water and sediment	3.5–4.0	90	Illumina HiSeq	10,050,000	–	Menzel et al., 2015
	Parvati River, India	Water and microbial mat	7.1–7.4	93–52	Illumina GaIIx	78,891,278	–	Sangwan et al., 2015
	Unkeshwar, India	Water	7.3	50–60	Illumina HiSeq	848,096	212.87	Mehetre et al., 2016
Deep sea hidrotermal vent	Mid-Atlantic Ridge	Microbial mat	9.0–11.0	90	Illumina HiSeq	46,361	35	Brazelton and Baross, 2009
	Juan de Fuca Ridge	Sulfide chimney	2.0–3.0	316	Roche 454	308,034	71	Xie et al., 2011
	Guaymas Baisin, Gulf of California	Black-smoker chimney	6.0	190	Roche 454	512,83	196.38	He et al., 2013
	Juan de Fuca Ridge	Vent fluid	–	125	Roche 454	808,051	–	Anderson et al., 2014
Biogas reactor	Germany	Microbial mat	6.64–8.11	55	Roche 454	303,493	120.50	Rademacher et al., 2012
	Linköping, Sweden	Digester material	7.5–8.1	50–55	Roche 454	250,478	–	Sundberg et al., 2013
	Romania	Sludge	6.94–7.62	55	Ion Torrent PGM	300,000	–	Pap et al., 2015
Oil reservoir	Norwegian sea	Oil, water and gas	–	85	Roche 454	702,607	345	Kotlar et al., 2011
	Gippsland Basin, Australia	Formation water	7.2	120	Roche 454	–	–	Li et al., 2013a
Compost	São Paulo Zoo Park, Brazil	Compost material	7	65.8–67.2	Roche 454	3,167,044	836	Martins et al., 2013
Gold mine	South Africa	Fracture water	9.3	60	Sanger and Roche 454	500,008	–	Chivian et al., 2008

*In those studies comprising several samples, the total reads and size reflected is just the one of the sample with higher values*.

Development of NGS has greatly enhanced this approach. The most widely used platforms for this kind of analysis in high temperature environments are Illumina and Roche 454 (Table 2). Illumina currently offers the highest throughput per run and the lowest cost per-base (Liu et al., 2012), generating read lengths up to 300 bp. On the other hand, Roche 454 gives longer reads (1 kb maximum), which are easier to map to a reference genome; however it is more expensive and has lower throughput (van Dijk et al., 2014). Even though they have substantial differences (Kumar et al., 2015), some studies have demonstrated that the information recovered from both sequencing platforms is comparable when analyzing the biodiversity of the same sample (Luo et al., 2012).

The main limitations of shotgun metagenome sequencing include its relatively expensive setup cost and the requirement of very high computing power for data storage, retrieval, and analysis. Another important drawback of this approach is that high quality whole community DNA is needed, which makes the extraction a critical step in the process of generating metagenomic data. Therefore, some studies have focused on the improvement of metagenomic DNA extraction from thermal environments (Mitchell and Takacs-Vesbach, 2008; Li et al., 2013a; Gupta et al., 2016). Nowadays the NGS platforms allow sequencing with low inputs of DNA, nevertheless in some cases it is necessary to amplify the metagenomic DNA to obtain enough quantity for preparing the sequencing libraries. As an example, Nakai et al. (2011) used multiple displacement amplification with Phi29 to sequence the metagenome of the hydrothermal fluid of the Mariana Trough, an active back-arc basin in the western Pacific Ocean. This amplification step is frequently required to generate viral metagenomic libraries, introducing a subsequent bias (Kim and Bae, 2011), as the extraction of enough high quality viral nucleic acids is a difficult process that usually relies on virus concentration methods.

To assess the taxonomic diversity with the short metagenomic reads obtained after sequencing, there are several non-exclusive approximations that can be done: analyzing taxonomically informative marker genes, grouping sequences into defined taxonomic groups (binning) or/and assembling sequences into definite genomes (Sharpton, 2014).

As mentioned before, the most frequently used taxonomically informative marker genes are rRNA genes or protein-coding genes that tend to be single copy and common to microbial genomes. In this approach, those reads that are homologs to the marker gene are identified in the sequences of the metagenome and annotated using sequence or phylogenetic similarity to the marker gene database sequences. Bioinformatics applications for this purpose include MetaPhyler (Liu et al., 2010), EMIRGE (Miller et al., 2011), and AMPHORA (Wu and Scott, 2012). Gladden et al. (2011) used EMIRGE to reconstruct near full-length small subunit (SSU) rRNA genes from metagenomic Illumina sequences to determine the taxonomy of compost-derived microbial consortia adapted to switchgrass at 60°C, finding a low-diversity community with predominance of *Rhodothermus marinus* and *Thermus thermophilus*. In another study, Klatt et al. (2011) used AMPHORA to identify the phylogenetic and functional marker genes in the assemblies of several hot springs cyanobacterial metagenomes from YNP. These studies allowed the discovery of novel chlorophototrophic bacteria belonging to uncharacterized lineages within the order Chlorobiales and within the Kingdom Chloroflexi. In a similar approach, Lin et al. (2015) and Colman et al. (2016) used a 16S rRNA gene-based diversity method blasting the metagenomic reads against the SILVA reference database to characterize bacterial populations in Shi-Huang-Ping acidic hot spring (Taiwan) and in two thermal springs in YNP, respectively.

Taxonomic binning is defined as the process of grouping reads or contigs and assigning them to operational taxonomic units, depending on information such as sequence similarity, sequence composition or read coverage (Dröge and McHardy, 2012). Metagenomic sequences can be binned based on their sequence similarity to a database of taxonomically annotated sequences using tools like MEGAN (Huson et al., 2011) or MG-RAST, a public resource for the automatic phylogenetic and functional analysis of metagenomes (Meyer et al., 2008). MEGAN bases its taxonomic classification on the NCBI taxonomy using BLAST. With this tool, Klatt et al. (2013) assessed the community structure of six phototrophic microbial mat communities in YNP and Badhai et al. (2015) revealed the dominance of Bacteria over Archaea in four geothermal springs in Odisha, India. Taxonomic binning can be done with assembled or unassembled reads, although assessing taxonomic abundance with assembled data can led to a miscalculation of the abundance of some taxa, as contigs are treated as a single sequence in most downstream analysis, hindering the analytical tools to accurately quantify the abundance of the taxon (Sharpton, 2014).

Assembly is described as the process of merging individual metagenomic reads into longer pieces of contiguous sequences (contigs) based on overlapping sequences and paired read information (Dröge and McHardy, 2012). Bioinformatic implements like MetaVelvet (Namiki et al., 2012) or IDBA-UD (Peng et al., 2012) have been used in the assembly of whole shotgun metagenome reads to study the taxonomical composition of different high-temperature environments. For example, MetaVelvet was applied in the study of eight globally distributed hot springs by Menzel et al. (2015) and IDBA-UD in the analysis of the community composition of Sungai Klah hot spring in Malaysia (Chan et al., 2015). This step can simplify bioinformatic analysis, but it may also produce chimeras, therefore researchers often bin reads and assemble each bin independently to decrease the probability of generating chimeras (Sharpton, 2014).

In recent studies, the integration of assembly and taxonomic binning by sequence composition allowed the reconstruction of several partial genomes from high-temperature environments such as the genome of a novel archaeal Rudivirus obtained from a Mexican hot spring, (Servín-Garcidueñas et al., 2013) or the draft genome sequence of *Thermoanaerobacter* sp. strain A7A, reconstructed from the metagenome of a 102°C hydrocarbon reservoir in the Bass Strait, Australia (Li et al., 2013b). Using a similar approach, Sangwan et al. (2015), reconstructed the genome of the bacterial predator *Bdellovibrio* ArHS, with the metagenomic assembly of the microbial mats of an arsenic rich hot spring in the Parvati river valley (Manikaran, India). Also, Sharma et al. (2016) combining genomic and metagenomic data, used two *Cellulosimicrobium cellulans* genomes derived from metagenomics, to study the evolution of pathogenicity across the species of *C. cellulans*.

## Functional analysis of thermophiles

### Sequence-based function prediction

The metagenomic reads obtained from shotgun sequencing of an environmental DNA can be annotated with functions to determine the functional diversity of the microbial community. This usually comprises two steps: identifying metagenomic reads that contain protein coding sequences (gene prediction), and comparing the coding sequences to a database of genes, proteins, protein families, or metabolic pathways (gene annotation) (Sharpton, 2014). Some frequently used databases for functional annotation are the SEED annotation system (Overbeek et al., 2014), the KEGG orthology (KO) database (Kanehisa et al., 2016) or the Pfam database, based on hidden Markov models (HMM) to classify in accordance with the protein domains (Finn et al., 2015). There are several robust web resources that can be easily used to perform gene prediction, database search, family classification, and annotation, including MG-RAST (Meyer et al., 2008), IMG/M (Markowitz et al., 2014), or SUPER-FOCUS (Silva et al., 2015). Considerable functional profiles of thermophilic populations have been based on these tools such as the study of the microbiota of Tuwa hot spring in India (Mangrola et al., 2015a) in which the functional annotation was performed using the MG-RAST pipeline. In this study, a high number of annotated features were classified as unknown function, suggesting the potential source of novel microbial species and their products. Similar results were found in the metagenome of Unkeshwar, another hot spring in India, where pathway annotation was done using KEGG (Mehetre et al., 2016). For each contig sequence, the assignment of KO numbers obtained from known reference hits was done, revealing up to 20% unclassified sequences. These results reflect a promising world of undiscovered proteins that could be explored to find new catalysts for biotechnological applications.

In this approach, it is important to consider that, despite the information given by functional annotation of the metagenomic sequences; the presence of a gene on a metagenome does not mean that it is expressed. Therefore, functional metagenomics, metatranscriptomics, and metaproteomics assays are necessary to assess the real community functional activity. To increase the probability of finding active functional genes involved in a substrate uptake and transformation, some studies use a substrate-induced enrichment of the community before the metagenomic DNA extraction. After, these genes can be detected either by sequence (Graham et al., 2011; Wang et al., 2016) or by functional metagenomics (Chow et al., 2012). Using this procedure, Graham et al. (2011) found and characterized an hyperthermophilic cellulase in an archaeal community, obtained by growth at 90°C of the sediment of a geothermal source enriched with crystalline cellulose.

Another important limitation of shotgun metagenomics is that the databases may be subjected to phylogenetic biases, as some communities are more accurately or more exhaustively annotated than others (Chistoserdova, 2010).

### Functional metagenomics

**Figure 2:**
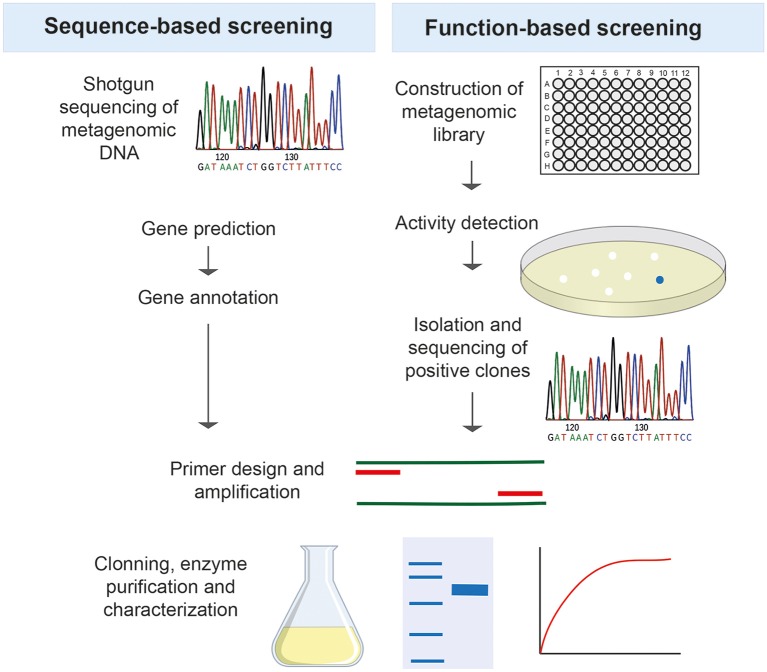
**The two major strategies used for screening metagenomes in search of new thermozymes**.

Function-based metagenomics relies on the construction of metagenomic libraries by cloning environmental DNA into expression vectors and propagating them in the appropriate hosts, followed by activity-based screening. After an active clone is identified, the sequence of the clone is determined, the gene of interest is amplified and cloned with the subsequent expression and characterization of the product to explore its biotechnological potential (Figure 2). This technique has the advantage of not requiring the cultivation of the native microorganisms or previous sequence information of known genes, thus representing a valuable approach for mining enzymes with new features.

The use of functional metagenomics allows the discovery of novel enzymes whose functions would not be predicted based on DNA sequence. This approach complements sequence-based metagenomics as the information from function-based analyses can be used to annotate genomes and metagenomes derived exclusively from sequence-based analyses (Lam et al., 2015). Therefore, several investigations in thermal environments combine sequencing methods (taxonomical and functional characterization) with functional screening of clones (Chen et al., 2007; Wemheuer et al., 2013; Leis et al., 2015; López-López et al., 2015b).

Depending on the size of the insert, functional metagenomics can be explored using fosmids (35–45 kb insert), BACs (~200 kb insert), cosmids (30–42 kb insert), or plasmids (<10 kb insert). Bigger inserts are more likely to contain complete genes and operons, allowing the expression of more enzymes. A great number of high temperature functional metagenomics studies use the commercial vector pCC1FOS (Table 3), which allows inserts up to 40 kb, and it is available in a toolkit to simplify the library construction. More information about this vector is compiled in Lam et al. (2015) review.

**Table 3:** **Examples of thermozymes obtained by functional metagenomics**.

**Family**	**Activity**	**Metagenomic DNA source**	**Vector**	**Host**	**Substrate**	**Total No. clones**	**Positive clones**	**References**
Lypolitic enzymes	Lipase	Biomass sequencing fed-batch reactor	pIAFS2	*E. coli* LE392MP	Tributyrin	10,000	10	Meilleur et al., 2009
	Lipase	Oil field soil	pZErO-2	*E. coli* TOP10	Tributyrin, rhodamine B	83,000	1	Fan et al., 2011
	Lipase	Botanical Garden soil, Hamburg, Germany	pSuperCos	*E. coli* EPI100	Tributyrin	6500	2	Chow et al., 2012
	Lipase	Industrial wastewater treatment plant	pBeloBAC11	*E. coli* DH10B	Tributyrin, rhodamine B	40,000	12	Sharma et al., 2012
	Lipase	Activated sludge	pUC18	*E. coli* DH5α	Tricaprylin	130,000	1	Roh and Schmid, 2013
	Lipase/Esterase	Hot spring, Kamchatka Peninsula	pCR-XL-TOPO	*E. coli* DH5α	Tributyrin	–	3	Wemheuer et al., 2013
	Lipase/Esterase	Oil polluted mud flat	pUC19	*E. coli* DH5α	Glyceryl trioctanoate	3000	2	Kim et al., 2015
	Esterase	Poly(DL-lactic acid) disks in compost	pUC18	*E. coli* DH10B	PLA solution	40,000	7	Mayumi et al., 2008
	Esterase	Compost	pCC1FOS	*E. coli* EPI300T1R	Tributyrin, gum arabic	13,000	10	Kang et al., 2011
	Esterase	Turpan Basin soil, China	pUC118	*E. coli* TOP10	Caprylate	21,000	3	Fan et al., 2012a
	Esterase	Turpan Basin soil, China	pUC118	*E. coli* DH5α	Caprylate	26,000	1	Fan et al., 2012b
	Esterase	Activated sludge	pBluescript SK+	*E. coli* DH5α	Tributyrin	40,000	1	Shao et al., 2013
	Esterase	Turpan Basin soil, China	pUC118	*E. coli* DH5α	Tributyrin	200,000	19	Wang et al., 2013
	Esterase	Deep-sea hydrothermal field, East Pacific	pUC18	*E. coli* XL1-blue	Tributyrin	–	–	Zhu et al., 2013
	Esterase	Guayas Basin smoker chimmeney	pCC2FOS	*E. coli* EPI300T1R	Tributyrin	18,000	7	Fu et al., 2015
	Esterase	Oil polluted mud flat	pUC19	*E. coli* DH5α	Tributyrin	3000	7	Kim et al., 2015
	Esterase	Hot spring, Furnas, Azores	pCT3FK	*T. thermophilus* BL03	Tributyrin	7968	6	Leis et al., 2015
	Esterase	Hot spring water Lobios, Galicia, Spain	pCC1FOS	*E. coli* EPI300T1R	Tributyrin	11,600	6	López-López et al., 2015b
Glycosidase	Cellulase	Compost	pCC2FOS	*E. coli* EPI300T1R	CMC Trypan blue	12,380	2	Kwon et al., 2010
	Cellulase	Biogas plant and elephant feces	pCC1FOS	*E. coli* EPI300T1R	CMC Congo red	29,000	14	Ilmberger et al., 2012
	Cellulase	Activated sludge	pUC18	*E. coli* DH5α	CMC	32,000	4	Sharma et al., 2012
	β-glucosidase	Hot spring water, mud and sediment	pCR-XL-TOPO	*E. coli* TOP10	Esculin hydrate, ferricammonium citrate	–	1	Schröder et al., 2014
	β-glucosidase	Thermophilic methanogenic digester	pCC1FOS	*E. coli* EPI300T1R	Esculin hydrate, ferricammonium citrate	–	–	Wang et al., 2015
	β-glucosidase	Termite hindgut	pUC118	*E. coli* DH5α	Esculin hydrate, ferricammonium citrate	800,000	13	Gao et al., 2016
	β-galactosidase	Hot spring, Hymalaya, India	pSMART LCamp	*E. coli* DH10B	X-gal	10,000	1	Gupta et al., 2012
	β-galactosidase	Turpan Basin soil, China	pUC19ΔlacZ	*E. coli* DH5α	X-gal	8000	1	Zhang et al., 2013
	β-galactosidase	Turpan Basin soil, China	pUC19ΔlacZ	*E. coli* DH5α	X-gal	700,000	1	Wang et al., 2014
	Xylanase	Compost	pCC2FOS	*E. coli* EPI300T1R	Xylan	12,380	5	Kwon et al., 2010
	Endoxylanase	Compost-soil	p18 GFP	*E. coli* DH10B	RBB-xylan	180,000	1	Verma et al., 2013
	β-xylosidase	Thermophilic methanogenic digester	pCC1FOS	*E. coli* EPI300T1R	Esculin hydrate, ferricammonium citrate	–	–	Wang et al., 2015
	Various	Raw and torrified wheat straw	pCC1FOS	*E. coli* EPI300T1R	X-fuc, X-gal, X-Xyl, X-Man, X-cel, X-glu	44,000	71	Maruthamuthu et al., 2016
Amylase	α-amylase	Western Ghats soil	pCC1FOS	*E. coli* EPI300T1R	Potassium iodide solution	76,000	1	Vidya et al., 2011
	α-amylase	Biogas reactor	pBK-CMV	*E. coli* XLOLR	AZCL-amylose	2000	1	Jabbour et al., 2013
Phosphatase	Phytase	Soil	pCC1FOS	*E. coli* EPI300T1R	Phytate	14,000	28	Tan et al., 2014
Protease	Serine protease	Sand Gobi desert	pCC1FOS	*E. coli* EPI300T1R	Skim milk	17,000	16	Neveu et al., 2011
	Serine protease	Sand Death Valley desert	pBSKII+	*E. coli* DH10B	Skim milk	30,000	1	Neveu et al., 2011
	Serine protease	Soil	pHT01	*E. coli* DH10B	AZCL-casein	–	1	Biver et al., 2013
	Patatin-like protein	Hot spring, Kamchatka	pCR-XL-TOPO	*E. coli* DH5α	Skim milk	–	1	Wemheuer et al., 2013
	Serine protease	Hot spring, Chumathang Ladakh	pUC18	*E. coli* DH5α	Casein	9000	1	Singh et al., 2015
Oxidoreductase	Extradiol dioxygenase	Activated sludge	pCC1FOS	*E. coli* EPI300T1R	Catechol	96,000	91	Suenaga et al., 2007
		Oil reservoir	pCC2FOS	*E. coli* EPI300T1R	Hexadecane	5000	72	de Vasconcellos et al., 2010
	Dioxygenase	Activated sludge	pBeloBAC11	*E. coli* DH10B	Phenol, catechol	40,000	4	Sharma et al., 2012

There are several technically challenging steps in library construction. Mainly, the high quality and length of the metagenomic DNA required for proceeding to the ligation into the vector and the need of obtaining a high proportion of clones in order to cover all the variability of the microbial community. This limitation is particularly important in soil studies, where it has been reported that contaminants like humic acids are present in metagenomic DNA extracts, interfering with the subsequent enzymatic reactions. Therefore, the widely extended method of soil DNA extraction established by Zhou et al. (1996), is usually accomplished with further purification of the sample that can lead to a loss of DNA yield. Some studies show that the humic acids can be easily removed by gel electrophoresis of the metagenomic DNA followed by gel extraction, as humic acids migrate faster than the large metagenomic DNA (Kwon et al., 2010). This simple approach was used to construct a Turpan Basin soil metagenomic library for a functional screening of thermostable beta-galactosidases (Wang et al., 2014). Alternatively, to avoid contaminating the circulating buffer, electrophoresis can be paused after humic acids have formed a front, excising the part of the gel containing the humic acids, and replacing it with fresh gel (Cheng et al., 2014).

Another important drawback that compromises the functional metagenomics approach is the selection of the expression host. Although the commonly used *E. coli* strains have relaxed requirements for promoter recognition and translation initiation, some genes from environmental samples may not be efficiently expressed due to differences in codon usage, transcription and/or translation initiation signals, protein-folding elements, post-translational modifications, or toxicity of the active enzyme (Uchiyama and Miyazaki, 2009). This problem could be even worse when the proteins expressed need special conditions to be active, such as high temperatures, considering that mesophiles, like *E. coli*, do not survive at these high temperature conditions. Accordingly, an alternative expression host may be required to overcome the heterologous expression of some genes derived from hot environments and thus, identify a broader range of enzymes. The thermophilic bacterium *T. thermophilus* has been proposed as a good candidate for function-based detection of thermozymes. In a recent functional screening to detect esterases, Leis et al. (2015) constructed two large insert fosmid metagenomic libraries of compost and hot spring water using pCT3FK, a pCC1FOS derived *T. thermophilus/E. coli* shuttle fosmid (Angelov et al., 2009), in *T. thermophilus* and compared them to the same libraries expressed in *E. coli*. Only two esterases were found at 60°C in the libraries generated in *E. coli* while 5 different esterases were discovered in the same libraries expressed in *T. thermophilus*. Therefore, this could be a suitable system to improve the detection of metagenome-derived thermozymes. The main restriction of this approach is that pCT3FK integrates into *T. thermophilus* chromosomal DNA. In fact, the genomes of the positive clones isolated by Leis et al. (2015) were completely sequenced before proceeding with the PCR amplification and cloning of the candidate genes, with the consequent cost of time and money. Other versatile broad-host-range cosmids that have been used in a soil study (pJC8 and pJC24) allow the phenotypic screening of the library in bacteria such as *Bacillus* and in the yeast *Saccharomyces* (Cheng et al., 2014). The selection of the appropriate substrate for the functional screening is also a crucial step in this approach, as the substrate may cause biases in the selection of the activities of interest. Recent studies suggest that the initial selection of active clones with general substrates should be followed by a more specific one to improve the effectiveness of the detection (Ferrer et al., 2016). Other biases and limitations of functional metagenomics and strategies for its improvement have been previously reviewed by Ferrer et al. (2005) and Ekkers et al. (2012).

Some hot environments where function-based screening of microbial communities have been done include hot springs (López-López et al., 2015b), deserts (Neveu et al., 2011), petroleum reservoirs (de Vasconcellos et al., 2010), or human-made environments like a biogas plant (Ilmberger et al., 2012), demonstrating the potential of functional metagenomics as a very important source of new thermozymes.

## Metagenome-derived thermozymes

Many industrial processes require elevated temperatures to take place. Thus, microorganisms surviving at temperatures above 55°C represent an important source of biotechnological richness for high temperature bioprocesses by producing a large variety of biocatalysts. Biotechnological processes carried out at high temperatures provide numerous benefits such as higher solubility of reagents, and reduced risk of microbial contamination (Mirete et al., 2016). From an industrial point of view, thermozymes possess certain advantages over their mesophilic counterparts as they are active and efficient under high temperatures, extreme pH values, high substrate concentrations, and high pressure (Sarmiento et al., 2015). Some of them are also highly resistant to denaturing agents and organic solvents (Fan et al., 2011; Roh and Schmid, 2013). In addition, thermozymes are easier to separate from heat-labile proteins during purification steps as reported by Pessela et al. (2004). As a result, high temperature-active enzymes can be potentially used in diverse industrial and biotechnological applications including food, paper and textile processing, chemical synthesis and the production of pharmaceuticals.

Some thermostable enzymes are still recovered by isolation from thermophilic microorganisms (Shi et al., 2013; Fuciños et al., 2014; Sen et al., 2016), however metagenomics has opened a new important field in the discovery of novel biocatalysts and has been revealed as a promising mining strategy of resources for the biotechnological and pharmaceutical industry. There are two different ways of screening a metagenome in search of thermozymes: a sequence-based approach and a function-based approach (Figure 2).

Sequence-based screening methods rely on the prior knowledge of conserved sequences of domains/proteins/families of interest. It involves primer designing followed by amplification and cloning of the metagenomic genes. The main drawback of this approach is its failure to detect fundamentally different novel genes, as it cannot discover non-homologous enzymes. Some potential biocatalysts have been isolated mining metagenomic sequences in prospecting for genes coding thermozymes (Table 4). Namely, a gene encoding a thermostable pectinase was isolated from a soil metagenome sample collected from hot springs of Manikaran (India), using a PCR-based cloning strategy with primers designed based on known sequences of pectinase genes from other species (Singh et al., 2012). The recombinant protein is proposed to be of great use in industrial processes due to its activity over a broad pH range. Thanks to this search based on sequence homology to related gene families, 22 putative ORFs (open reading frames) were identified from a switchgrass-adapted compost community finding a bi-functional β-xylosidase/α-arabinofuranosidase that maintained ~75% of its activity after 16 h at 60°C (Dougherty et al., 2012). The same sequence-based approach was used by Ferrandi et al. (2015) who discovered, cloned and characterized two novel limonene-1,2-epoxide hydrolases (LEHs) with an *in-silico* screening of the LEHs sequences in the assembled contigs from hot spring metagenomes.

**Table 4:** **Examples of thermozymes isolated by sequence-based screening of metagenomes**.

**Enzyme**	**Source of metagenomic DNA**	**References**
Cellulase	Long-term dry thermophilic methanogenic digester	Wang et al., 2015
Xylanase	Cow dung compost	Sun et al., 2015
Xylanase	Long-term dry thermophilic methanogenic digester	Wang et al., 2015
Endoxylanase	Compost	Dougherty et al., 2012
β-galactosidase	Hot spring water, Yongtai	Liu et al., 2015
β-xylosidase/α-arabinofuranosidase	Compost	Dougherty et al., 2012
β-xylosidase	Long-term dry thermophilic methanogenic digester	Wang et al., 2015
Pectinase	Hot spring soil, Manikaran, India	Singh et al., 2012
α-fucosidase	Compost	Dougherty et al., 2012
Phytase (phosphatase)	Insect-cultivated fungus gardens	Tan et al., 2016
Limonene 1,2-epoxide hydrolase	Hot springs water	Ferrandi et al., 2015
Nitrilase	Atlantis II Deep Brine Pool, Red Sea	Sonbol et al., 2016
α-amylase	Hydrotermal vent, Juan de la Fuca	Wang et al., 2011
Dehidroclorinase	Soil contaminated with HCH-isomers	Macwan et al., 2011
Polymerase	Hot spring 3173 Pol	Moser et al., 2012

The function-based metagenomic screening is the most important way to discover novel thermozymes as it doesn't rely on the sequence. The main advantage of directly screening for enzymatic activities from metagenome libraries is that it gives access to previously unknown genes and their encoded enzymes. Thus, some completely new thermozymes that couldn't be found by sequence screening have been discovered, like the unusual glycosyltransferase-like enzyme with β-galactosidase activity recovered by Wang et al. (2013) from a Turpan Basin soil metagenomic library. Function-based metagenomic screening has allowed the discovery of a wide range of thermozymes (Table 3). In this review, we focus on the recovery of the functional-derived thermostable metagenomic enzymes that are mostly used in biocatalysis and industrial sectors, such as lipolytic enzymes, glycosidases, proteases, and oxidoreductases (Böhnke and Perner, 2015).

### Lipolytic enzymes

Lipolytic enzymes, comprising esterases (EC 3.1.1.1) and lipases (EC 3.1.1.3), are extensively distributed in microorganisms, plants, and animals. They catalyze the hydrolysis, synthesis, or transesterification of ester bonds. At present, these enzymes represent about 20% of commercialized enzymes for industrial use (López-López et al., 2015a), as they have great potential in several industrial processes such as production of biodegradable polymers, detergents, food flavoring, oil biodegradation, or waste treatment, among others (Anobom et al., 2014). Therefore, a considerable number of functional metagenomics studies are focused on mining thermal environments in search for these enzymes (Table 3).

Lipases are generally defined as carboxylesterases hydrolyzing water-insoluble (acyl chain length >10) triglycerides, with trioleoylglycerol as the standard substrate. In contrast, esterases catalyze the hydrolysis of short-chain esters (acyl chain length <10) with tributylglycerols (tributyrin) as the standard substrate, although lipases are also capable of hydrolyzing esterase substrates (Rhee et al., 2005). At least 200 different substrates have been successfully applied in assays for functional selection of esterase/lipase biocatalysts in metagenomic clone libraries (Ferrer et al., 2016), including the widely used tributyrin (Rhee et al., 2005; Meilleur et al., 2009; López-López et al., 2015b), and p-nitrophenyl (NP) acetate (Wang et al., 2013). Meilleur et al. (2009) isolated a new alkali-thermostable lipase with an optimal activity at 60°C and pH 10.5 by functional screening of a metagenomic cosmid library from the biomass produced in a gelatin enriched fed-batch reactor. Another gene coding for a thermostable esterase was detected by functional screening of fosmid environmental DNA libraries constructed with metagenomes from thermal environmental samples of Indonesia (Rhee et al., 2005). The recombinant esterase was active from 30 up to 95°C with an optimal pH of approximately 6.0. Mayumi et al. (2008), generated a metagenomic library with the community DNA extracted from biodegradable polyester poly(lactic acid) (PLA) disks buried in compost and found a PLA depolymerase that had an esterase domain. Purified enzyme showed the highest activity at 70°C and degraded not only PLA, but also various aliphatic polyesters, tributyrin, and p-NP esters. As mentioned before, those enzymes able of retaining activity even in the presence of organic solvents are considered very interesting for industrial applications. A new thermophilic organic solvent-tolerant and halotolerant esterase with an optimum pH and temperature of 7.0 and 50°C, respectively, was found in the functional screening of a soil metagenomic library with 48,000 clones (Wang et al., 2013).

Apart from these above cited sources, metagenomic esterases, and lipases have been isolated by functional screening of other hot environments like deep-sea hydrothermal vents (Zhu et al., 2013) and hot springs (López-López et al., 2015b) as shown in Table 3. A more extensive review of metagenome derived extremophilic lipolytic enzymes can be found in López-López et al. (2014).

### Glycosidases

The enzymes that hydrolyze glycosidic bonds between two or more sugars or a sugar and a nonsugar moiety within carbohydrates or oligosaccharides are known as glycosyl hydrolases (GHs) or glycosidases (Sathya and Khan, 2014). There are 115 GH families, collected in the Carbohydrate Active enZyme database (CAZy; http://www.cazy.org) (Lombard et al., 2014), including a broad number of enzymes like cellulases, β-galactosidases, amylases, and pectinases.

Cellulases encompass a group of complex enzymes conformed by endo-β-1,4 glucanases, cellobiohydrolases, cellodextrinases, and β-glucosidases. These enzymes work together to degrade cellulose into simple sugars and their thermostable representatives could be used in biofuel production from lignocellulosic biomass (Bhalla et al., 2013). Several substrates can be employed in plate-based screens for the functional detection of clones harboring cellulase activity, such as carboxymethyl-cellulose in combination with trypan blue, Gram's iodine, or Congo Red. Meddeb-Mouelhi et al. (2014), found that Gram's iodine may lead to the identification of false positives, making Congo Red a more suitable dye for this approach. Using Congo Red dye as a colorimetric substrate, Ilmberger et al. (2012) obtained two fosmid clones derived from a carboxymethyl-cellulose (CMC)-enriched library from a biogas plant. These two fosmids were designated as pFosCelA2 and pFosCelA3, encoding two thermostable cellulases with significant activities in the presence of 30% (v/v) ionic liquids (ILs). This is an interesting property for the cellulose degradation, as cellulose could increase its solubility in the ILs.

From the group of cellulases, β-glucosidases have attracted considerable attention in recent years due to their important roles in various biotechnological processes such as hydrolysis of isoflavone glucosides or the production of fuel ethanol from agricultural residues (Singhania et al., 2013). Other uses of β-glucosidases include the cleavage of phenolic and phytoestrogen glucosides from fruits and vegetables for medical applications or to enhance the quality of beverages. An archaeal β-glucosidase (Bgl1) showing activity toward cellobiose, cellotriose, and lactose was isolated from a metagenome from a hydrothermal spring in the island of Sγo Miguel (Azores, Portugal) (Schröder et al., 2014).

β-Galactosidases (EC 3.2.1.23), which hydrolyze lactose to glucose and galactose, have two main applications in the food industry: the production of low-lactose milk and dairy products for lactose intolerant people and the generation of galactooligosaccharides from lactose by the transgalactosylation reaction. These enzymes can be also used in the revalorisation of cheese whey (Becerra et al., 2015), a by-product of the dairy industry with a high organic load that can be considered a pollutant.

The most widely used substrate for the β-galactosidase screening, 5-bromo-4-chloro-3-indolyl-β-D-galactopyranoside (X-gal), is the substrate providing, in some cases, the lowest number of positive hits in relation to the total number of clones screened (Ferrer et al., 2016). Usually, the positive clones capable of hydrolyzing the X-gal are further tested against ortho-NP-β-galactoside (ONPG) and lactose (Wierzbicka-Woś et al., 2013). Mayor drawbacks for the use of β-galactosidase in industrial processes is the inhibition by the reaction products, leading to a decrease in the reaction rates or even to stop the enzymatic reaction completely. The thermostable β-galactosidase (Gal308) discovered by Zhang et al. (2013) exhibited high tolerance to galactose and glucose with the highest activity at 78°C, an optimum pH of 6.8 and high enzyme activity with lactose as substrate. The authors suggest that these properties would make it a good candidate for the production of low-lactose milk and dairy products. Another novel and thermostable alkalophilic β-D-galactosidase with an optimum temperature at 65°C and with high transglycosylation activity was identified through functional screening of a metagenomic library from a hot spring in northern Himalayan region of India (Gupta et al., 2012).

Xylans, made of β-1,4 linked xylopyranoses as a linear backbone with branches, constitute the second most significant group of polysaccharides in plant cell walls and are degraded by xylanases (Sathya and Khan, 2014). These hemicellulolytic enzymes are mostly used as biobleaching agents in the paper and pulp industry. The discovery of thermostable and alkali-stable xylanases has become an important goal in this field since this process requires high temperatures and alkali media, but this is not the only application of thermostable xylanases (Kumar et al., 2016). Functional metagenomics of hot environments represents an interesting source of xylanases. As an example, a novel alkali-stable and thermostable GH-11 endoxylanase encoding gene (*Mxyl*), was isolated by functional screening of a compost-soil metagenome (Verma et al., 2013). The thermostability of this enzyme was subsequently engineered by directed site mutagenesis (Verma and Satyanarayana, 2013).

Amylases are known as enzymes that catalyze the hydrolysis of starch into sugars (Sundarram et al., 2014). A novel and thermostable amylase with the highest activity at 90°C was retrieved from a black smoker chimney by combining fosmid library construction with pyrosequencing (Wang et al., 2011). Another α-amylase was isolated in the functional screening of a metagenomic library of Western Ghats soil constructed in pCC1FOS. This amylase retained 30% activity after incubation for 60 min at 80°C and had an optimal pH of 5.0 and could be potentially used in some industrial processes like liquefaction and saccharification of starch in food industry, or formulation of enzymatic detergents and removing starch from textiles (Vidya et al., 2011).

### Proteases

Proteases are protein-hydrolyzing enzymes classified into acidic, neutral, or alkaline groups, based on their optimum pH. They can also be classified into aspartic, cysteine, glutamic, metallo, serine, and threonine protease types based on the amino acids present in their active sites (Singh et al., 2015). These enzymes are widely used in various industries such as detergent, food, and leather (Haddar et al., 2009; George et al., 2014). A thermotolerant, alkali-stable and oxidation resistant protease (CHpro1) was found by functional screening of a metagenomic library constructed from sediments of hot springs in Chumathang area of Ladakh, India (Singh et al., 2015). This enzyme, that showed optimum activity at pH 11 and stability in high alkaline range, could be especially interesting for the detergent industry, as the pH of laundry detergents is generally in the range of 9.0–12.0. This property, in addition to the resistance in the presence of detergent compounds, like oxidizing agents, and the possibility of working at high wash temperatures (optimum activity at 80°C), makes it a very suitable detergent protease.

### Oxidoreductases

These enzymes catalyze oxidation-reduction reactions, in which hydrogen or oxygen atoms or electrons are transferred between molecules and are important biocatalysts for several industrial processes. From a pharmaceutical point of view, oxidoreductases can act like quorum-quenching enzymes, degrading signal molecules to block quorum-sensing-dependent infection, as reported by Bijtenhoorn et al. (2011), who found a soil-derived dehydrogenase/reductase implicated in the decreasing of *Pseudomonas aeruginosa* biofilm formation and virulence of *Caenorhabditis elegans*. These enzymes can also be used in food industry as they catalyze oxidation- reduction reactions that can play an important role in taste, flavor and nutritional value of aliments such as virgin olive oil (Peres et al., 2015). Another relevant application of oxidoreductases is their role in decomposing specific recalcitrant contaminants by precipitation or by transforming them to other products, leading to a better final treatment of the waste. Some oxidoreductases that can be used for this purpose include peroxidases, polyphenol oxidases, and estradiol dioxygenases (EDOs) (Durán and Esposito, 2000). Suenaga et al. (2007) constructed a metagenomic library from activated sludge used to treat coke plant wastewater containing various organic pollutants like phenol, mono- and polycyclic nitrogen-containing aromatics or aromatic hydrocarbons, among others. The library was screened for EDOs, using catechol as a substrate, yielding 91 EDO-positive clones, 38 of them were sequenced in order to conduct similarity searches using BLASTX. A polyphenol oxidase enzyme, with alkaline laccase activity and highly soluble expression, showing the optimum activity of 55°C, was isolated from a functional screening of DNA from mangrove soil (Ye et al., 2010).

## Comparative metagenomics

The increasing number of metagenomes from high-temperature environments sequenced and the possibility of generating more sequences with a lower cost of time and money has enabled the comparison of metagenomic sequences between and within environments, opening a new field in metagenomics. Comparative metagenomics can enlighten how the microbial community taxa or the metabolic potential vary between sampling locations or time points, as well as explain the influence of several factors, such as high temperatures, in the taxonomical and functional composition of an ecosystem. Comparison of metagenomic data recovered from different high temperature habitats indicates that these communities are different with respect to species abundance and microbial composition. However, some groups of species are more commonly represented, for example, bacterial taxa such as *Thermotoga, Deinococcus-Thermus*, and *Proteobacteria*, as well as Archaea, like *Methanococcus, Thermoprotei*, and *Thermococcus* (Lewin et al., 2013). The comparison between metagenomes derived from six distantly located hot springs of varying temperature and pH revealed a wide distribution of four archaeal viral families, *Ampullaviridae, Bicaudaviridae, Lipothrixviridae*, and *Rudiviridae* (Gudbergsdóttir et al., 2016). Even though the important role of viruses in high temperature ecosystems has been demonstrated, the comparative studies are limited since the diversity of thermophilic viruses in many hot environments remains unknown, as revealed by Adriaenssens et al. (2015) in the Namib desert hypoliths metagenome, where the majority of the viral sequence reads were classified as unknown.

Comparative metagenomics can also increase our insight into the adaptation of microorganisms to high temperature environments. Xie et al. (2011) compared the sequences obtained from a fosmid metagenomic library of a black smoker chimney 4143-1 in the Mothra hydrothermal vent field at the Juan de Fuca Ridge with metagenomes of different environments, including a biofilm of a carbonate chimney from the Lost City hydrothermal vent field (90°C, pH 9–11 fluids). This study revealed that the deep-sea vent chimneys are highly enriched in genes for mismatch repair and homologous recombination, and exhibited a high proportion of transposases. These enzymes, which are critical in horizontal gene transfer, were also abundantly found when comparing the metagenomic data obtained from three different deep-sea hydrothermal vent chimneys (He et al., 2013). This fact supports the previous hypothesis that horizontal gene transfer may be common in the deep-sea vent chimney biosphere and could be an important source of phenotypic diversity (Brazelton and Baross, 2009).

Other comparative studies show that, apart from temperature, pH is also an important factor in the composition of microbial communities. A comparison of the biodiversity and community composition in eight geographically remote hot springs (temperature range between 61 and 92°C and pH between 1.8 and 7) showed a decrease in biodiversity with increasing temperature and decreasing pH (Menzel et al., 2015). The loss of biodiversity in hot environments with low pH was also observed by Song et al. (2013), showing a more diverse bacterial population in non-acidic hot springs than in acidic hot springs from the Yunan Province (China).

IMG/M (Markowitz et al., 2014) and MG-RAST (Meyer et al., 2008) are two frequently used metagenomics pipelines to easily perform comparative analysis of microbial communities, and can be explored to find sequences of different high temperature environments, since a considerable number of metagenomes are deposited in their databases. MG-RAST has about 240 thousand data sets containing over 800 billion sequences and more than 36 thousand public metagenomes, including 225 metagenomes (0.61%) from different thermophilic biomes with temperatures ranging from 52 to 122°C obtained by whole shotgun and/or amplicon sequencing (data publicly available at MG-RAST server on August 2016).

Usually, these studies require statistical tools to explore multivariate data, like principal component analysis (PCA), in order to compare and contrast metagenomes from different environments. PCA is one of the most widely used statistical analyses for genomic data as it is a simple and robust data reduction technique that can be applied to large data sets. A more exhaustive description of some of the statistical analysis that can be used to compare metagenomes can be found in the study by Dinsdale et al. (2013). In this study, the metabolic functions of 212 metagenomes, including six different hot springs, were compared between and within environments using different statistical methods. Several tools like STAMP (statistical analysis of metagenomics profiles, Parks et al., 2014) and PRIMER-E can be used for this purpose, allowing the statistical analysis of multivariate data.

## Future perspectives

### High-throughput screening methods

Although the new ultra-fast sequencing technologies quickly generate a remarkable number of target gene candidates, functional assays are still needed to confirm them. These assays for protein function represent one of the most reliable and invaluable tools for mining target genes. Thus, developing of high-throughput screening (HTS) methods and improved chromogenic substrates for the detection of thermozymes (Kračun et al., 2015) is a priority for reducing the time invested in primary screening. HTS techniques increase the success of function-based metagenomic screens since they compensate for the often low hit rates in such screens (Ekkers et al., 2012). Apart from conventional high throughput screens, which use microtiter plate wells to store a large number of clones (Ko et al., 2013), microarray-based technologies coupled with microfluidic devices, cell compartmentalization, flow cytometry, and cell sorting are arising as promising new technologies for this purpose (Najah et al., 2014; Meier et al., 2015; Vidal-Melgosa et al., 2015). Microfluidic technologies are of undeniable interest when it comes to reaching screening rates of a million clones per day (Ufarté et al., 2015). This screening method generally uses fluorogenic substrates (Najah et al., 2013) and it is based on the encapsulation of single clones of the metagenomic library in droplets, followed by the substrate induced gene-expression screening and the fluorescence-activated cell sorting to isolate plasmidic clones containing the genes of interest (Colin et al., 2015; Hosokawa et al., 2015). The main advantages of this ultra-fast screening method are the small volume required (usually picoliters to femtoliters) and the capability of detecting intracellular, extracellular, and membrane proteins. This approach could be used for the screening of thermozymes, as droplets can be incubated at high temperature before proceeding to the screening and fluorescence sorting. In this regard, there is an ongoing FP7 Marie Curie Action named HOTDROPS that involves four companies and four academic partners (including the authors' group) aimed to develop a microfluidics-based ultra-high-throughput platform for the selection of thermozymes from metagenomics and directed evolution libraries.

### Advanced sequencing

Until recently, most of the sequences collected in reference databases were related to humans and their pathogens. Currently, the advances in sequencing technologies have enabled the generation of considerable amounts of longer reads in less time. This fact, in addition to the lower per base cost and the development of metagenomics, has produced a relevant increase in the number of genomes sequenced and annotated deposited in databases like GenBank, thus covering a high range of microorganisms from a wide variety of habitats, including high temperature environments. Therefore, the bias in the databases toward microorganisms with clinical or pathogenic interest is decreasing, allowing a better analysis of the populations with metagenomics. Furthermore, metagenomics is becoming a tool in reach of many laboratories with the recent release of new cheaper and smaller devices such as the Oxford Nanopore MinION, a USB flash drive-size sequencer that measures deviations in electrical current as a single DNA strand passes through a protein nanopore (Bayley, 2015). However, this technology presents high error rates compared to the others (Goodwin et al., 2015) and still has to be improved.

Altogether, these breakthrough developments make metagenomics a more affordable and robust tool to explore the taxonomy and the functional diversity of microbial communities. Nevertheless, the complexity of microbial species, together with the limitations of the technology to cover fully whole genome sequences, still pose a great challenge for metagenome research. NGS technologies have limitations and remain at least an order of magnitude more expensive than other conventional microbiological assays, thus samples often must be individually barcoded and pooled into single runs to decrease costs. All these deficiencies will probably disappear as technologies continue developing like they did in the last years, from the end of the human genome sequencing project in 2003 (Collins et al., 2003), up to now.

### Advances in bioinformatics

Due to the massive amount of metagenome data generated in the last 10 years, infrastructural developments associated with managing and serving sequence data are needed. Additionally, the fast growth in the size of data complicates its storage, organization, and distribution. As the volume of metagenomics data keeps growing, new assemblers have been developed, namely MEGAHIT that can assemble large and complex metagenomics data in a time and cost-efficient way, especially on a single-node server (Li et al., 2015).

New bioinformatic pipelines designed to support researchers involved in functional and taxonomic studies of environmental microbial communities have been released like BioMaS (Fosso et al., 2015), DUDes (Piro et al., 2016), or MOCAT2 (Kultima et al., 2016), among others.

Since there is an increasing number of complex communities sequenced, improved statistical methodology is needed, especially to enhance comparative studies where a large number of covariates (e.g., environmental or host physiological parameters) are collected for each sample.

## Author contributions

MD did all the data gathering and write-up. ER and MG supervised and reviewed the manuscript, providing comments and guidance during the manuscript development.

## Funding

Funding both from the European Union Seventh Framework Programme (FP7/2007-2013) under grant agreement n° 324439, and from the Xunta de Galicia (Consolidación D.O.G. 10-10-2012, Contract Number: 2012/118) co-financed by FEDER. The work of MD was supported by a FPU fellowship (Ministerio de Educación Cultura y Deporte) FPU12/05050.

### Conflict of interest statement

The authors declare that the research was conducted in the absence of any commercial or financial relationships that could be construed as a potential conflict of interest.

## References

[B1] Acinas S. G. Marcelino L. A. Klepac-Ceraj V. Polz M. F. (2004). Divergence and redundancy of 16S rRNA sequences in genomes with multiple rrn operons. J. Bacteriol. 186, 2629–2635. 10.1128/JB.186.9.2629-2635.2004 15090503 PMC387781

[B2] Adriaenssens E. M. Van Zyl L. De Maayer P. Rubagotti E. Rybicki E. Tuffin M.. (2015). Metagenomic analysis of the viral community in namib desert hypoliths. Environ. Microbiol. 17, 480–495. 10.1111/1462-2920.12528 24912085

[B3] Anderson R. E. Brazelton W. J. Baross J. A. Altschul S. Gish W. Miller W.. (2011). Using CRISPRs as a metagenomic tool to identify microbial hosts of a diffuse flow hydrothermal vent viral assemblage. FEMS Microbiol. Ecol. 77, 120–133. 10.1111/j.1574-6941.2011.01090.x 21410492

[B4] Anderson R. E. Sogin M. L. Baross J. A. Anderson R. Beltrán M. Hallam S.. (2014). Evolutionary strategies of viruses, bacteria and archaea in hydrothermal vent ecosystems revealed through metagenomics. PLoS ONE 9:e109696. 10.1371/journal.pone.0109696 25279954 PMC4184897

[B5] Angelov A. Mientus M. Liebl S. Liebl W. (2009). A two-host fosmid system for functional screening of (meta)genomic libraries from extreme thermophiles. Syst. Appl. Microbiol. 32, 177–185. 10.1016/j.syapm.2008.01.003 19285378

[B6] Anobom C. D. Pinheiro A. S. De-Andrade R. A. Aguieiras E. C. G. Andrade G. C. Moura M. V.. (2014). From structure to catalysis: recent developments in the biotechnological applications of lipases. Biomed Res. Int. 2014:684506. 10.1155/2014/684506 24783219 PMC3982246

[B7] Ashelford K. E. Chuzhanova N. A. Fry J. C. Jones A. J. Weightman A. J. (2005). At least 1 in 20 16S rRNA sequence records currently held in public repositories is estimated to contain substantial anomalies. Appl. Environ. Microbiol. 71, 7724–7736. 10.1128/AEM.71.12.7724-7736.2005 16332745 PMC1317345

[B8] Badhai J. Ghosh T. S. Das S. K. (2015). Taxonomic and functional characteristics of microbial communities and their correlation with physicochemical properties of four geothermal springs in Odisha, India. Front. Microbiol. 6:1166. 10.3389/fmicb.2015.01166 26579081 PMC4620158

[B9] Baker G. Gaffar S. Cowan D. Suharto R. (2001). Bacterial community analysis of Indonesian hot springs. FEMS Microbiol. Lett. 200, 103–109. 10.1016/S0378-1097(01)00207-5 11410357

[B10] Barns S. M. Fundyga R. E. Jeffries M. W. Pace N. R. (1994). Remarkable archaeal diversity detected in a Yellowstone National Park hot spring environment. Proc. Natl. Acad. Sci. U.S.A. 91, 1609–1613. 10.1073/pnas.91.5.1609 7510403 PMC43212

[B11] Bayley H. (2015). Nanopore sequencing: from imagination to reality. Clin. Chem. 61, 25–31. 10.1373/clinchem.2014.223016 25477535 PMC4404466

[B12] Becerra M. Cerdán M. E. González-Siso M. I. (2015). Biobutanol from cheese whey. Microb. Cell Fact. 14, 27. 10.1186/s12934-015-0200-1 25889728 PMC4404668

[B13] Bhalla A. Bansal N. Kumar S. Bischoff K. M. Sani R. K. (2013). Bioresource technology improved lignocellulose conversion to biofuels with thermophilic bacteria and thermostable enzymes. Bioresour. Technol. 128, 751–759. 10.1016/j.biortech.2012.10.145 23246299

[B14] Bijtenhoorn P. Mayerhofer H. Müller-Dieckmann J. Utpatel C. Schipper C. Hornung C.. (2011). A novel metagenomic short-chain dehydrogenase/reductase attenuates *Pseudomonas aeruginosa* biofilm formation and virulence on *Caenorhabditis elegans*. PLoS ONE 6:e26278. 10.1371/journal.pone.0026278 22046268 PMC3202535

[B15] Biver S. Portetelle D. Vandenbol M. (2013). Characterization of a new oxidant-stable serine protease isolated by functional metagenomics. Springerplus 2:410. 10.1186/2193-1801-2-410 24024096 PMC3765597

[B16] Böhnke S. Perner M. (2015). A function-based screen for seeking RubisCO active clones from metagenomes: novel enzymes influencing RubisCO activity. ISME J. 9, 735–745. 10.1038/ismej.2014.163 25203835 PMC4331584

[B17] Brady A. L. Sharp C. E. Grasby S. E. Dunfield P. F. (2015). Anaerobic carboxydotrophic bacteria in geothermal springs identified using stable isotope probing. Front. Microbiol. 6:897. 10.3389/fmicb.2015.00897 26388850 PMC4555085

[B18] Brazelton W. J. Baross J. A. (2009). Abundant transposases encoded by the metagenome of a hydrothermal chimney biofilm. ISME J. 3, 1420–1424. 10.1038/ismej.2009.79 19571895

[B19] Breitbart M. Wegley L. Leeds S. Schoenfeld T. Rohwer F. (2004). Phage community dynamics in hot springs. Appl. Environ. Microbiol. 70, 1633–1640. 10.1128/AEM.70.3.1633-1640.2004 15006788 PMC368299

[B20] Brock T. D. Freeze H. (1969). Thermus aquaticus gen. n. and sp. n., a nonsporulating extreme thermophile. J. Bacteriol. 98, 289–297. 5781580 10.1128/jb.98.1.289-297.1969 PMC249935

[B21] Cai L. Ye L. Tong A. H. Y. Lok S. Zhang T. Handelsman J.. (2013). Biased diversity metrics revealed by bacterial 16S pyrotags derived from different primer sets. PLoS ONE 8:e53649. 10.1371/journal.pone.0053649 23341963 PMC3544912

[B22] Chan C. S. Chan K.-G. Tay Y.-L. Chua Y.-H. Goh K. M. (2015). Diversity of thermophiles in a Malaysian hot spring determined using 16S rRNA and shotgun metagenome sequencing. Front. Microbiol. 6:177. 10.3389/fmicb.2015.00177 25798135 PMC4350410

[B23] Chen Z. W. Liu Y. Y. Wu J. F. She Q. Jiang C. Y. Liu S. J. (2007). Novel bacterial sulfur oxygenase reductases from bioreactors treating gold-bearing concentrates. Appl. Microbiol. Biotechnol. 74, 688–698. 10.1007/s00253-006-0691-0 17111141

[B24] Cheng J. Pinnell L. Engel K. Neufeld J. D. Charles T. C. (2014). Versatile broad-host-range cosmids for construction of high quality metagenomic libraries. J. Microbiol. Methods 99, 27–34. 10.1016/j.mimet.2014.01.015 24495694

[B25] Chistoserdova L. (2010). Recent progress and new challenges in metagenomics for biotechnology. Biotechnol. Lett. 32, 1351–1359. 10.1007/s10529-010-0306-9 20495950

[B26] Chivian D. Brodie E. L. Alm E. J. Culley D. E. Dehal P. S. DeSantis T. Z.. (2008). Environmental genomics reveals a single-species ecosystem deep within Earth. Science 322, 275–278. 10.1126/science.1155495 18845759

[B27] Chow J. Kovacic F. Dall Antonia Y. Krauss U. Fersini F. Schmeisser C.. (2012). The metagenome-derived enzymes LipS and LipT increase the diversity of known lipases. PLoS ONE 7:e47665. 10.1371/journal.pone.0047665 23112831 PMC3480424

[B28] Cole J. R. Wang Q. Fish J. A. Chai B. McGarrell D. M. Sun Y.. (2014). Ribosomal database project: data and tools for high throughput rRNA analysis. Nucleic Acids Res. 42, 1–10. 10.1093/nar/gkt1244 24288368 PMC3965039

[B29] Colin P.-Y. Kintses B. Gielen F. Miton C. M. Fischer G. Mohamed M. F.. (2015). Ultrahigh-throughput discovery of promiscuous enzymes by picodroplet functional metagenomics. Nat. Commun. 6:10008. 10.1038/ncomms10008 26639611 PMC4686663

[B30] Collins F. S. Morgan M. Patrinos A. Watson J. D. Olson M. V. Collins F. S.. (2003). The human genome project: lessons from large-scale biology. Science 300, 286–290. 10.1126/science.1084564 12690187

[B31] Colman D. R. Jay Z. J. Inskeep W. P. Jennings R. deM. Maas K. R. Rusch D. B.. (2016). Novel, deep-branching heterotrophic bacterial populations recovered from thermal spring metagenomes. Front. Microbiol. 7:304. 10.3389/fmicb.2016.00304 27014227 PMC4791363

[B32] Coyotzi S. Pratscher J. Murrell J. C. Neufeld J. D. (2016). Targeted metagenomics of active microbial populations with stable-isotope probing. Curr. Opin. Biotechnol. 41, 1–8. 10.1016/j.copbio.2016.02.017 26946369

[B33] Dadheech P. K. Glöckner G. Casper P. Kotut K. Mazzoni C. J. Mbedi S.. (2013). Cyanobacterial diversity in the hot spring, pelagic and benthic habitats of a tropical soda lake. FEMS Microbiol. Ecol. 85, 389–401. 10.1111/1574-6941.12128 23586739

[B34] De la Torre J. R. Walker C. B. Ingalls A. E. Könneke M. Stahl D. A. (2008). Cultivation of a thermophilic ammonia oxidizing archaeon synthesizing crenarchaeol. Environ. Microbiol. 10, 810–818. 10.1111/j.1462-2920.2007.01506.x 18205821

[B35] Denonfoux J. Parisot N. Dugat-Bony E. Biderre-Petit C. Boucher D. Morgavi D. P.. (2013). Gene capture coupled to high-throughput sequencing as a strategy for targeted metagenome exploration. DNA Res. 20, 185–196. 10.1093/dnares/dst001 23364577 PMC3628448

[B36] Dinsdale E. A. Edwards R. A. Bailey B. A. Tuba I. Akhter S. McNair K.. (2013). Multivariate analysis of functional metagenomes. Front. Genet. 4:41. 10.3389/fgene.2013.00041 23579547 PMC3619665

[B37] Dougherty M. J. D'haeseleer P. Hazen T. C. Simmons B. A. Adams P. D. Hadi M. Z. (2012). Glycoside hydrolases from a targeted compost metagenome, activity-screening and functional characterization. BMC Biotechnol. 12:38. 10.1186/1472-6750-12-38 22759983 PMC3477009

[B38] Dröge J. McHardy A. C. (2012). Taxonomic binning of metagenome samples generated by next-generation sequencing technologies. Brief. Bioinform. 13, 646–655. 10.1093/bib/bbs031 22851513

[B39] Durán N. Esposito E. (2000). Potential applications of oxidative enzymes and phenoloxidase-like compounds in wastewater and soil treatment: a review. Appl. Catal. B Environ. 28, 83–99. 10.1016/S0926-3373(00)00168-5

[B40] Ekkers D. M. Cretoiu M. S. Kielak A. M. Van Elsas J. D. (2012). The great screen anomaly-a new frontier in product discovery through functional metagenomics. Appl. Microbiol. Biotechnol. 93, 1005–1020. 10.1007/s00253-011-3804-3 22189864 PMC3264863

[B41] Fan X. Liu X. Huang R. Liu Y. (2012a). Identification and characterization of a novel thermostable pyrethroid-hydrolyzing enzyme isolated through metagenomic approach. Microb. Cell Fact. 11:33. 10.1186/1475-2859-11-33 22409882 PMC3317823

[B42] Fan X. Liu X. Liu Y. (2012b). The cloning and characterization of one novel metagenome-derived thermostable esterase acting on N-acylhomoserine lactones. J. Mol. Catal. B Enzym. 83, 29–37. 10.1016/j.molcatb.2012.07.006

[B43] Fan X. Liu X. Wang K. Wang S. Huang R. Liu Y. (2011). Highly soluble expression and molecular characterization of an organic solvent-stable and thermotolerant lipase originating from the metagenome. J. Mol. Catal. B Enzym. 72, 319–325. 10.1016/j.molcatb.2011.07.009

[B44] Fancello L. Trape S. Robert C. Boyer M. Popgeorgiev N. Raoult D.. (2012). Viruses in the desert: a metagenomic survey of viral communities in four perennial ponds of the Mauritanian Sahara. ISME J. 7, 359–369. 10.1038/ismej.2012.101 23038177 PMC3554411

[B45] Farmer J. (1998). Thermophiles, early biosphere evolution, and the origin of life on Earth: implications for the exobiological exploration of Mars. J. Geophys. Res. 103, 457–461. 10.1029/98JE01542

[B46] Ferrandi E. E. Sayer C. Isupov M. N. Annovazzi C. Marchesi C. Iacobone G.. (2015). Discovery and characterization of thermophilic limonene-1,2-epoxide hydrolases from hot spring metagenomic libraries. FEBS J. 282, 2879–2894. 10.1111/febs.13328 26032250

[B47] Ferrer M. Martínez-Abarca F. Golyshin P. N. (2005). Mining genomes and “metagenomes” for novel catalysts. Curr. Opin. Biotechnol. 16, 588–593. 10.1016/j.copbio.2005.09.001 16171989

[B48] Ferrer M. Martínez-Martínez M. Bargiela R. Streit W. R. Golyshina O. V. Golyshin P. N. (2016). Estimating the success of enzyme bioprospecting through metagenomics: current status and future trends. Microb. Biotechnol. 9, 22–34. 10.1111/1751-7915.12309 26275154 PMC4720405

[B49] Ferris M. J. Kühl M. Wieland A. Ward D. M. (2003). Cyanobacterial ecotypes in different optical microenvironments of a 68⋅C hot spring mat community revealed by 16S-23S rRNA internal transcribed spacer region variation. Appl. Environ. Microbiol. 69, 2893–2898. 10.1128/AEM.69.5.2893-2898.2003 12732563 PMC154543

[B50] Fiala G. Stetter K. O. (1986). Pyrococcus furiosus sp. nov. represents a novel genus of marine heterotrophic archaebacteria growing optimally at 100⋅C. Arch. Microbiol. 145, 56–61. 10.1007/BF00413027

[B51] Finn R. D. Coggill P. Eberhardt R. Y. Eddy S. R. Mistry J. Mitchell A. L.. (2015). The Pfam protein families database: towards a more sustainable future. Nucleic Acids Res. 44, D279–D285. 10.1093/nar/gkv1344 26673716 PMC4702930

[B52] Fosso B. Santamaria M. Marzano M. Alonso-Alemany D. Valiente G. Donvito G.. (2015). BioMaS: a modular pipeline for Bioinformatic analysis of Metagenomic AmpliconS. BMC Bioinformatics 16:203. 10.1186/s12859-015-0595-z 26130132 PMC4486701

[B53] Fu L. He Y. Xu F. Ma Q. Wang F. Xu J. (2015). Characterization of a novel thermostable patatin - like protein from a Guaymas basin metagenomic library. Extremophiles 19, 829–840. 10.1007/s00792-015-0758-x 26016814

[B54] Fuciños P. Atanes E. López-López O. Solaroli M. Cerdán M. E. González-Siso M. I. (2014). Cloning, expression, purification and characterization of an oligomeric His-tagged thermophilic esterase from *Thermus thermophilus* HB27. Process Biochem. 49, 927–935. 10.1016/j.procbio.2014.03.006

[B55] Gao G. Wang A. Gong B. Li Q. Liu Y. He Z.. (2016). A novel metagenome-derived gene cluster from termite hindgut : encoding phosphotransferase system components and high glucose tolerant glucosidase. Enzyme Microb. Technol. 84, 24–31. 10.1016/j.enzmictec.2015.12.005 26827771

[B56] George N. Singh P. Kumar V. Puri N. Gupta N. (2014). Approach to ecofriendly leather : characterization and application of an alkaline protease for chemical free dehairing of skins and hides at pilot scale. J. Clean. Prod. 79, 249–257. 10.1016/j.jclepro.2014.05.046

[B57] Gerbl F. W. Weidler G. W. Wanek W. Erhardt A. Stan-Lotter H. (2014). Thaumarchaeal ammonium oxidation and evidence for a nitrogen cycle in a subsurface radioactive thermal spring in the Austrian Central Alps. Front. Microbiol. 5:225. 10.3389/fmicb.2014.00225 24904540 PMC4032944

[B58] Ghelani A. Patel R. Mangrola A. Dudhagara P. (2015). Cultivation-independent comprehensive survey of bacterial diversity in Tulsi Shyam Hot Springs, India. Genomics Data 4, 54–56. 10.1016/j.gdata.2015.03.003 26484176 PMC4536058

[B59] Gladden J. M. Allgaier M. Miller C. S. Hazen T. C. VanderGheynst J. S. Hugenholtz P.. (2011). Glycoside hydrolase activities of thermophilic bacterial consortia adapted to switchgrass. Appl. Environ. Microbiol. 77, 5804–5812. 10.1128/AEM.00032-11 21724886 PMC3165268

[B60] Goodwin S. Gurtowski J. Ethe-Sayers S. Deshpande P. Schatz M. McCombie W. R. (2015). Oxford Nanopore sequencing and *de novo* assembly of a eukaryotic genome. Genome Res. 25, 1750–1756. 10.1101/013490 26447147 PMC4617970

[B61] Graham J. E. Clark M. E. Nadler D. C. Huffer S. Chokhawala H. A. Rowland S. E.. (2011). Identification and characterization of a multidomain hyperthermophilic cellulase from an archaeal enrichment. Nat. Commun. 2:375. 10.1038/ncomms1373 21730956

[B62] Gudbergsdóttir S. R. Menzel P. Krogh A. Young M. Peng X. (2016). Novel viral genomes identified from six metagenomes reveal wide distribution of archaeal viruses and high viral diversity in terrestrial hot springs. Environ. Microbiol. 18, 863–874. 10.1111/1462-2920.13079 26439881

[B63] Gupta P. Manjula A. Rajendhran J. Gunasekaran P. Vakhlu J. (2016). Comparison of metagenomic DNA extraction methods for soil sediments of high elevation Puga hot spring in Ladakh, India to explore bacterial diversity. Geomicrobiol. J. 10.1080/01490451.2015.1128995. [Epub ahead of print].

[B64] Gupta R. Govil T. Capalash N. Sharma P. (2012). Characterization of a glycoside hydrolase family 1 β-galactosidase from hot spring metagenome with transglycosylation activity. Appl. Biochem. Biotechnol. 168, 1681–1693. 10.1007/s12010-012-9889-z 23015191

[B65] Haddar A. Agrebi R. Bougatef A. Hmidet N. Sellami-kamoun A. Nasri M. (2009). Bioresource technology two detergent stable alkaline serine-proteases from Bacillus mojavensis A21 : purification, characterization and potential application as a laundry detergent additive. Bioresour. Technol. 100, 3366–3373. 10.1016/j.biortech.2009.01.061 19269812

[B66] Handelsman J. (2004). Metagenomics : application of genomics to uncultured microorganisms. Microbiol. Mol. Biol. Rev. 68, 669–685. 10.1128/MBR.68.4.669 15590779 PMC539003

[B67] He Y. Xiao X. Wang F. (2013). Metagenome reveals potential microbial degradation of hydrocarbon coupled with sulfate reduction in an oil-immersed chimney from Guaymas Basin. Front. Microbiol. 4:148. 10.3389/fmicb.2013.00148 23785357 PMC3682177

[B68] Hedlund B. P. Dodsworth J. A. Cole J. K. Panosyan H. H. (2013). An integrated study reveals diverse methanogens, Thaumarchaeota, and yet-uncultivated archaeal lineages in Armenian hot springs. Antonie van Leeuwenhoek 104, 71–82. 10.1007/s10482-013-9927-z 23632917

[B69] Hosokawa M. Hoshino Y. Nishikawa Y. Hirose T. Yoon D. H. Mori T.. (2015). Droplet-based microfluidics for high-throughput screening of a metagenomic library for isolation of microbial enzymes. Biosens. Bioelectron. 67, 379–385. 10.1016/j.bios.2014.08.059 25194237

[B70] Huang Q. Jiang H. Briggs B. R. Wang S. Hou W. Li G.. (2013). Archaeal and bacterial diversity in acidic to circumneutral hot springs in the Philippines. FEMS Microbiol. Ecol. 85, 452–464. 10.1111/1574-6941.12134 23607726

[B71] Hug K. Maher W. A. Stott M. B. Krikowa F. Foster S. Moreau J. W. (2014). Microbial contributions to coupled arsenic and sulfur cycling in the acid-sulfide hot spring Champagne Pool, New Zealand. Front. Microbiol. 5:569. 10.3389/fmicb.2014.00569 25414696 PMC4220137

[B72] Huson D. Mitra S. Ruscheweyh H. (2011). Integrative analysis of environmental sequences using MEGAN4. Genome Res. 21, 1552–1560. 10.1101/gr.120618.111 21690186 PMC3166839

[B73] Ilmberger N. Meske D. Juergensen J. Schulte M. Barthen P. Rabausch U.. (2012). Metagenomic cellulases highly tolerant towards the presence of ionic liquids - Linking thermostability and halotolerance. Appl. Microbiol. Biotechnol. 95, 135–146. 10.1007/s00253-011-3732-2 22143172

[B74] Inskeep W. P. Jay Z. J. Herrgard M. J. Kozubal M. A. Rusch D. B. Tringe S. G.. (2013). Phylogenetic and functional analysis of metagenome sequence from high-temperature archaeal habitats demonstrate linkages between metabolic potential and geochemistry. Front. Microbiol. 4:95. 10.3389/fmicb.2013.00095 23720654 PMC3654217

[B75] Inskeep W. P. Rusch D. B. Jay Z. J. Herrgard M. J. Kozubal M. A. Richardson T. H.. (2010). Metagenomes from high-temperature chemotrophic systems reveal geochemical controls on microbial community structure and function. PLoS ONE 5:e9773. 10.1371/journal.pone.0009773 20333304 PMC2841643

[B76] Jabbour D. Sorger A. Sahm K. (2013). A highly thermoactive and salt-tolerant α -amylase isolated from a pilot-plant biogas reactor. Appl. Microbiol. Biotechnol. 97, 2971–2978. 10.1007/s00253-012-4194-x 22743714 PMC3602641

[B77] Kanehisa M. Sato Y. Kawashima M. Furumichi M. Tanabe M. (2016). KEGG as a reference resource for gene and protein annotation. Nucleic Acids Res. 44, D457–D462. 10.1093/nar/gkv1070 26476454 PMC4702792

[B78] Kang C.-H. Oh K.-H. Lee M.-H. Oh T.-K. Kim B. H. Yoon J.-H. (2011). A novel family VII esterase with industrial potential from compost metagenomic library. Microb. Cell Fact. 10:41. 10.1186/1475-2859-10-41 21619698 PMC3120640

[B79] Kim H. J. Jeong Y. S. Jung W. K. Kim S. K. Lee H. W. Kahng H. Y.. (2015). Characterization of novel family IV esterase and family I.3 lipase from an oil-polluted mud flat metagenome. Mol. Biotechnol. 57, 781–792. 10.1007/s12033-015-9871-4 25943044

[B80] Kim K. H. Bae J. W. (2011). Amplification methods bias metagenomic libraries of uncultured single-stranded and double-stranded DNA viruses. Appl. Environ. Microbiol. 77, 7663–7668. 10.1128/AEM.00289-11 21926223 PMC3209148

[B81] Klatt C. G. Inskeep W. P. Herrgard M. J. Jay Z. J. Rusch D. B. Tringe S. G.. (2013). Community structure and function of high-temperature chlorophototrophic microbial mats inhabiting diverse geothermal environments. Front. Microbiol. 4:106. 10.3389/fmicb.2013.00106 23761787 PMC3669762

[B82] Klatt C. G. Wood J. M. Rusch D. B. Bateson M. M. Hamamura N. Heidelberg J. F.. (2011). Community ecology of hot spring cyanobacterial mats: predominant populations and their functional potential. ISME J. 5, 1262–1278. 10.1038/ismej.2011.73 21697961 PMC3146275

[B83] Ko K. C. Han Y. Cheong D. E. Choi J. H. Song J. J. (2013). Strategy for screening metagenomic resources for exocellulase activity using a robotic, high-throughput screening system. J. Microbiol. Methods 94, 311–316. 10.1016/j.mimet.2013.07.010 23892060

[B84] Kotlar H. K. Lewin A. Johansen J. Throne-Holst M. Haverkamp T. Markussen S.. (2011). High coverage sequencing of DNA from microorganisms living in an oil reservoir 2.5 kilometres subsurface. Environ. Microbiol. Rep. 3, 674–681. 10.1111/j.1758-2229.2011.00279.x 23761356

[B85] Kozubal M. A. Romine M. Jennings R. deM. Jay Z. J. Tringe S. G. Rusch D. B.. (2013). Geoarchaeota: a new candidate phylum in the Archaea from high-temperature acidic iron mats in Yellowstone National Park. ISME J. 7, 622–634. 10.1038/ismej.2012.132 23151644 PMC3578567

[B86] Kračun S. K. Schückel J. Westereng B. Thygesen L. G. Monrad R. N. Eijsink V. G. H.. (2015). A new generation of versatile chromogenic substrates for high-throughput analysis of biomass-degrading enzymes. Biotechnol. Biofuels 8:70. 10.1186/s13068-015-0250-y 25969695 PMC4428106

[B87] Kultima J. R. Coelho L. P. Forslund K. Huerta-Cepas J. Li S. S. Driessen M.. (2016). MOCAT2: a metagenomic assembly, annotation and profiling framework. Bioinformatics 32, 2520–2523. 10.1093/bioinformatics/btw183 27153620 PMC4978931

[B88] Kumar S. Krishnani K. K. Bhushan B. Brahmane M. P. (2015). Metagenomics: retrospect and prospects in high throughput age. Biotechnol. Res. Int. 2015:121735. 10.1155/2015/121735 26664751 PMC4664791

[B89] Kumar V. Marín-Navarro J. Shukla P. (2016). Thermostable microbial xylanases for pulp and paper industries: trends, applications and further perspectives. World J. Microbiol. Biotechnol. 32, 34. 10.1007/s11274-015-2005-0 26754672

[B90] Kwon E. J. Jeong Y. S. Kim Y. H. Kim S. K. Na H. B. Kim J. (2010). Construction of a metagenomic library from compost and screening of cellulase- and xylanase-positive clones. J. Appl. Biol. Chem. 53, 702–708. 10.3839/jksabc.2010.106

[B91] Lam K. N. Cheng J. Engel K. Neufeld J. D. Charles T. C. (2015). Current and future resources for functional metagenomics. Front. Microbiol.6:1196. 10.3389/fmicb.2015.01196 26579102 PMC4625089

[B92] Leis B. Angelov A. Mientus M. Li H. Pham V. T. T. Lauinger B.. (2015). Identification of novel esterase-active enzymes from hot environments by use of the host bacterium *Thermus thermophilus*. Front. Microbiol. 6:275. 10.3389/fmicb.2015.00275 25904908 PMC4389547

[B93] Lewin A. Wentzel A. Valla S. (2013). Metagenomics of microbial life in extreme temperature environments. Curr. Opin. Biotechnol. 24, 516–525. 10.1016/j.copbio.2012.10.012 23146837

[B94] Li A. Chu Y. Wang X. Ren L. Yu J. Liu X.. (2013a). A pyrosequencing-based metagenomic study of methane-producing microbial community in solid-state biogas reactor. Biotechnol. Biofuels 6:3. 10.1186/1754-6834-6-3 23320936 PMC3618299

[B95] Li D. Greenfield P. Rosewarne C. P. Midgley J. (2013b). Draft genome sequence of Thermoanaerobacter sp. Strain A7A, reconstructed from a metagenome obtained from a high- temperature hydrocarbon reservoir in the Bass Strait, Australia. Genome Announc. 1, e00701-13. 10.1128/genomeA.00701-13 24029756 PMC3772140

[B96] Li D. Liu C.-M. Luo R. Sadakane K. Lam T.-W. (2015). MEGAHIT: an ultra-fast single-node solution for large and complex metagenomics assembly via succinct de Bruijn graph. Bioinformatics 31, 1674–1676. 10.1093/bioinformatics/btv033 25609793

[B97] Lin K.-H. Liao B.-Y. Chang H.-W. Huang S.-W. Chang T.-Y. Yang C.-Y.. (2015). Metabolic characteristics of dominant microbes and key rare species from an acidic hot spring in Taiwan revealed by metagenomics. BMC Genomics 16:1029. 10.1186/s12864-015-2230-9 26630941 PMC4668684

[B98] Liu B. Gibbons T. Ghodsi M. Pop M. (2010). MetaPhyler: taxonomic profiling for metagenomic sequences, 2010 IEEE International Conference on Bioinformatics and Biomedicine (BIBM) (Hong Kong), 95–100.

[B99] Liu L. Li Y. Li S. Hu N. He Y. Pong R.. (2012). Comparison of next-generation sequencing systems. J. Biomed. Biotechnol. 2012:251364. 10.1155/2012/251364 22829749 PMC3398667

[B100] Liu W. T. Marsh T. L. Cheng H. Forney L. J. (1997). Characterization of microbial diversity by determining terminal restriction fragment length polymorphisms of genes encoding 16S rRNA. Appl. Environ. Microbiol. 63, 4516–4522. 9361437 10.1128/aem.63.11.4516-4522.1997 PMC168770

[B101] Liu Z. Zhao C. Deng Y. Huang Y. Liu B. (2015). Characterization of a thermostable recombinant β-galactosidase from a thermophilic anaerobic bacterial consortium YTY-70. Biotechnol. Biotechnol. Equip. 29, 547–554. 10.1080/13102818.2015.1015244

[B102] Lombard V. Ramulu H. G. Drula E. Coutinho P. M. Henrissat B. (2014). The carbohydrate-active enzymes database (CAZy) in 2013. Nucleic Acids Res. 42, 490–495. 10.1093/nar/gkt1178 24270786 PMC3965031

[B103] López-López O. Cerdán M. E. González Siso M. I. (2014). New extremophilic lipases and esterases from metagenomics. Curr. Protein Pept. Sci. 15, 445–455. 10.2174/1389203715666140228153801 24588890 PMC4093774

[B104] López-López O. Cerdán M.-E. González-Siso M.-I. (2015a). *Thermus thermophilus* as a source of thermostable lipolytic enzymes. Microorganisms 3, 792–808. 10.3390/microorganisms3040792 PMC5023265 27682117

[B105] López-López O. Knapik K. Cerdán M. E. González-Siso M. I. (2015b). Metagenomics of an alkaline hot spring in Galicia (Spain): microbial diversity analysis and screening for novel lipolytic enzymes. Front. Microbiol. 6:1291. 10.3389/fmicb.2015.01291 26635759 PMC4653306

[B106] Luo C. Tsementzi D. Kyrpides N. Read T. Konstantinidis K. T. Nelson K.. (2012). Direct comparisons of Illumina vs. Roche 454 sequencing technologies on the same microbial community DNA sample. PLoS ONE 7:e30087. 10.1371/journal.pone.0030087 22347999 PMC3277595

[B107] Macwan A. S. Javed S. Kumar A. (2011). Isolation of a novel thermostable dehydrochlorinase (LinA) from a soil metagenome. 3 Biotech 1, 193–198. 10.1007/s13205-011-0012-x 22558537 PMC3339611

[B108] Mangrola A. Dudhagara P. Koringa P. Joshi C. G. Parmar M. Patel R. (2015a). Deciphering the microbiota of Tuwa hot spring, India using shotgun metagenomic sequencing approach. Genomics Data 4, 153–155. 10.1016/j.gdata.2015.04.014 26484204 PMC4535658

[B109] Mangrola A. V. Dudhagara P. Koringa P. Joshi C. G. Patel R. K. (2015b). Shotgun metagenomic sequencing based microbial diversity assessment of Lasundra hot spring, India. Genomics Data 4, 73–75. 10.1016/j.gdata.2015.03.005 26484181 PMC4536006

[B110] Manoharan L. Kushwaha S. K. Hedlund K. Ahrén D. (2015). Captured metagenomics: large-scale targeting of genes based on “sequence capture” reveals functional diversity in soils. DNA Res. 22, 451–460. 10.1093/dnares/dsv026 26490729 PMC4675713

[B111] Markowitz V. M. Chen I. M. A. Chu K. Szeto E. Palaniappan K. Pillay M.. (2014). IMG/M 4 version of the integrated metagenome comparative analysis system. Nucleic Acids Res. 42, 568–573. 10.1093/nar/gkt919 24136997 PMC3964948

[B112] Martins L. F. Antunes L. P. Pascon R. C. de Oliveira J. C. F. Digiampietri L. A. Barbosa D.. (2013). Metagenomic analysis of a tropical composting operation at the São Paulo zoo park reveals diversity of biomass degradation functions and organisms. PLoS ONE 8:e61928. 10.1371/journal.pone.0061928 23637931 PMC3637033

[B113] Maruthamuthu M. Jiménez D. J. Stevens P. Van Elsas J. D. (2016). A multi-substrate approach for functional metagenomics-based screening for (hemi) cellulases in two wheat straw- degrading microbial consortia unveils novel thermoalkaliphilic enzymes. BMC Genomics 17:86. 10.1186/s12864-016-2404-0 26822785 PMC4730625

[B114] Mayumi D. Akutsu-Shigeno Y. Uchiyama H. Nomura N. Nakajima-Kambe T. (2008). Identification and characterization of novel poly(DL-lactic acid) depolymerases from metagenome. Appl. Microbiol. Biotechnol. 79, 743–750. 10.1007/s00253-008-1477-3 18461319

[B115] Meddeb-Mouelhi F. Kelly J. Beauregard M. (2014). Enzyme and Microbial Technology A comparison of plate assay methods for detecting extracellular cellulase and xylanase activity. Enzyme Microb. Technol. 66, 16–19. 10.1016/j.enzmictec.2014.07.004 25248694

[B116] Mehetre G. T. Paranjpe A. S. Dastager S. G. Dharne M. S. (2016). Complete metagenome sequencing based bacterial diversity and functional insights from basaltic hot spring of Unkeshwar, Maharashtra, India. Genomics Data 7, 140–143. 10.1016/j.gdata.2015.12.031 26981391 PMC4778638

[B117] Meier M. J. Paterson E. S. Lambert I. B. (2015). Use of substrate-induced gene expression in metagenomic analysis of an aromatic hydrocarbon-contaminated soil. Appl. Environ. Microbiol. 82, 897–909. 10.1128/AEM.03306-15 26590287 PMC4725278

[B118] Meilleur C. Hupé J. F. Juteau P. Shareck F. (2009). Isolation and characterization of a new alkali-thermostable lipase cloned from a metagenomic library. J. Ind. Microbiol. Biotechnol. 36, 853–861. 10.1007/s10295-009-0562-7 19333634

[B119] Menzel P. Gudbergsdóttir S. R. Rike A. G. Lin L. Zhang Q. Contursi P.. (2015). Comparative metagenomics of eight geographically remote terrestrial hot springs. Microb. Ecol. 70, 411–424. 10.1007/s00248-015-0576-9 25712554

[B120] Meyer F. Paarmann D. D'souza M. Olson R. Glass E. Kubal M.. (2008). The metagenomics RAST server—a public resource for the automatic phylo- genetic and functional analysis of metagenomes. BMC Bioinformatics 9:386. 10.1186/1471-2105-9-386 18803844 PMC2563014

[B121] Miller C. S. Baker B. J. Thomas B. C. Singer S. W. Banfield J. F. Pace N.. (2011). EMIRGE: reconstruction of full-length ribosomal genes from microbial community short read sequencing data. Genome Biol. 12:R44. 10.1186/gb-2011-12-5-r44 21595876 PMC3219967

[B122] Mirete S. Morgante V. González-Pastor J. E. (2016). Functional metagenomics of extreme environments. Curr. Opin. Biotechnol. 38, 143–149. 10.1016/j.copbio.2016.01.017 26901403

[B123] Mitchell K. R. Takacs-Vesbach C. D. (2008). A comparison of methods for total community DNA preservation and extraction from various thermal environments. J. Ind. Microbiol. Biotechnol. 35, 1139–1147. 10.1007/s10295-008-0393-y 18633656

[B124] Morrison L. E. Tanner F. W. (1922). Studies on Thermophilic Bacteria: I. Aerobic Thermophilic Bacteria from Water. J. Bacteriol. 7, 343–366. 16558962 10.1128/jb.7.3.343-366.1922 PMC378975

[B125] Moser M. J. DiFrancesco R. A. Gowda K. Klingele A. J. Sugar D. R. Stocki S.. (2012). Thermostable DNA polymerase from a viral metagenome is a potent RT-PCR enzyme. PLoS ONE 7:e38371. 10.1371/journal.pone.0038371 22675552 PMC3366922

[B126] Muyzer G. De Waal E. C. Uitterlinden A. G. (1993). Profiling of complex microbial populations by denaturing gradient gel electrophoresis analysis of polymerase chain reaction-amplified genes coding for 16S rRNA. Appl. Env. Microbiol. 59, 695–700. 7683183 10.1128/aem.59.3.695-700.1993 PMC202176

[B127] Najah M. Calbrix R. Mahendra-Wijaya I. P. Beneyton T. Griffiths A. D. Drevelle A. (2014). Droplet-based microfluidics platform for ultra-high-throughput bioprospecting of cellulolytic microorganisms. Chem. Biol. 21, 1722–1732. 10.1016/j.chembiol.2014.10.020 25525991

[B128] Najah M. Mayot E. Mahendra-Wijaya I. P. Griffiths A. D. Ladame S. Drevelle A. (2013). New glycosidase substrates for droplet-based microfluidic screening. Anal. Chem. 85, 9807–9814. 10.1021/ac4022709 24079367

[B129] Nakai R. Abe T. Takeyama H. Naganuma T. (2011). Metagenomic analysis of 0.2-μm-passable microorganisms in deep-sea hydrothermal fluid. Mar. Biotechnol. 13, 900–908. 10.1007/s10126-010-9351-6 21279410

[B130] Namiki T. Hachiya T. Tanaka H. Sakakibara Y. (2012). MetaVelvet: an extension of Velvet assembler to *de novo* metagenome assembly from short sequence reads. Nucleic Acids Res. 40:e155. 10.1093/nar/gks678 22821567 PMC3488206

[B131] Neveu J. Regeard C. Dubow M. S. (2011). Isolation and characterization of two serine proteases from metagenomic libraries of the Gobi and Death Valley deserts. Appl. Microbiol. Biotechnol. 91, 635–644. 10.1007/s00253-011-3256-9 21494865

[B132] Olsen G. J. Lane D. J. Giovannoni S. J. Pace N. R. Stahl D. A. (1986). Microbial ecology and evolution: a ribosomal RNA approach. Annu. Rev. Microbiol. 40, 337–365. 10.1146/annurev.mi.40.100186.002005 2430518

[B133] Overbeek R. Olson R. Pusch G. D. Olsen G. J. Davis J. J. Disz T.. (2014). The SEED and the Rapid Annotation of microbial genomes using Subsystems Technology (RAST). Nucleic Acids Res. 42, 206–214. 10.1093/nar/gkt1226 24293654 PMC3965101

[B134] Panda A. K. Bisht S. S. Kumar N. S. De Mandal S. (2015). Investigations on microbial diversity of Jakrem hot spring, Meghalaya, India using cultivation-independent approach. Genomics Data 4, 156–157. 10.1016/j.gdata.2015.04.016 26484205 PMC4535621

[B135] Pap B. Györkei Á. Boboescu I. Z. Nagy I. K. Bíró T. Kondorosi É.. (2015). Temperature-dependent transformation of biogas-producing microbial communities points to the increased importance of hydrogenotrophic methanogenesis under thermophilic operation. Bioresour. Technol. 177, 375–380. 10.1016/j.biortech.2014.11.021 25481804

[B136] Parks D. H. Tyson G. W. Hugenholtz P. Beiko R. G. (2014). STAMP: statistical analysis of taxonomic and functional profiles. Bioinformatics 30, 3123–3124. 10.1093/bioinformatics/btu494 25061070 PMC4609014

[B137] Peng Y. Leung H. C. M. Yiu S. M. Chin F. Y. L. (2012). IDBA-UD: a *de novo* assembler for single-cell and metagenomic sequencing data with highly uneven depth. Bioinformatics 28, 1420–1428. 10.1093/bioinformatics/bts174 22495754

[B138] Peres F. Martins L. L. Ferreira-Dias S. (2015). Influence of enzymes and technology on virgin olive oil composition. Crit. Rev. Food Sci. Nutr. [Epub ahead of print]. 10.1080/10408398.2015.1092107 26466636

[B139] Pessela B. C. C. Torres R. Fuentes M. Mateo C. Filho M. Carrascosa A. V.. (2004). A simple strategy for the purification of large thermophilic proteins overexpressed in mesophilic microorganisms: application to multimeric enzymes from Thermus sp. strain T2 expressed in *Escherichia coli*. Biotechnol. Prog. 20, 1507–1511. 10.1021/bp049785t 15458336

[B140] Pham V. H. T. Kim J. (2012). Cultivation of unculturable soil bacteria. Trends Biotechnol. 30, 475–484. 10.1016/j.tibtech.2012.05.007 22770837

[B141] Piro V. C. Lindner M. S. Renard B. Y. (2016). DUDes: a top-down taxonomic profiler for metagenomics. Bioinformatics 32, 2272–2280. 10.1093/bioinformatics/btw150 27153591

[B142] Prokofeva M. I. Kublanov I. V. Nercessian O. Tourova T. P. Kolganova T. V. Lebedinsky A. V.. (2005). Cultivated anaerobic acidophilic/acidotolerant thermophiles from terrestrial and deep-sea hydrothermal habitats. Extremophiles 9, 437–448. 10.1007/s00792-005-0461-4 15970992

[B143] Purcell D. Sompong U. Yim L. C. Barraclough T. G. Peerapornpisal Y. Pointing S. B. (2007). The effects of temperature, pH and sulphide on the community structure of hyperthermophilic streamers in hot springs of northern Thailand. FEMS Microbiol. Ecol. 60, 456–466. 10.1111/j.1574-6941.2007.00302.x 17386034

[B144] Quast C. Pruesse E. Yilmaz P. Gerken J. Schweer T. Yarza P.. (2013). The SILVA ribosomal RNA gene database project: improved data processing and web-based tools. Nucleic Acids Res. 41, 590–596. 10.1093/nar/gks1219 23193283 PMC3531112

[B145] Rademacher A. Zakrzewski M. Schlüter A. Schönberg M. Szczepanowski R. Goesmann A.. (2012). Characterization of microbial biofilms in a thermophilic biogas system by high-throughput metagenome sequencing. FEMS Microbiol. Ecol. 79, 785–799. 10.1111/j.1574-6941.2011.01265.x 22126587

[B146] Rhee J.-K. Ahn D.-G. Kim Y.-G. Oh J.-W. (2005). New thermophilic and thermostable esterase with sequence similarity to the hormone-sensitive lipase family, cloned from a metagenomic library. Appl. Environ. Microbiol. 71, 817–825. 10.1128/AEM.71.2.817-825.2005 15691936 PMC546692

[B147] Roh C. Schmid R. D. (2013). Isolation of an organic solvent-tolerant lipolytic enzyme from uncultivated microorganism. Appl. Biochem. Biotechnol. 171, 1750–1758. 10.1007/s12010-013-0464-z 23996140

[B148] Rohwer F. Prangishvili D. Lindell D. (2009). Roles of viruses in the environment. Environ. Microbiol. 11, 2771–2774. 10.1111/j.1462-2920.2009.02101.x 19878268

[B149] Rozanov A. S. Bryanskaya A. V. Malup T. K. Meshcheryakova I. A. Lazareva E. V. Taran O. P.. (2014). Molecular analysis of the benthos microbial community in Zavarzin thermal spring (Uzon Caldera, Kamchatka, Russia). BMC Genomics 15:S12. 10.1186/1471-2164-15-S12-S12 25563397 PMC4303939

[B150] Sahm K. John P. Nacke H. Wemheuer B. Grote R. Daniel R.. (2013). High abundance of heterotrophic prokaryotes in hydrothermal springs of the Azores as revealed by a network of 16S rRNA gene-based methods. Extremophiles 17, 649–662. 10.1007/s00792-013-0548-2 23708551

[B151] Sangwan N. Lambert C. Sharma A. Gupta V. Khurana P. Khurana J. P.. (2015). Arsenic rich Himalayan hot spring metagenomics reveal genetically novel predator-prey genotypes. Environ. Microbiol. Rep. 7, 812–823. 10.1111/1758-2229.12297 25953741

[B152] Sarmiento F. Peralta R. Blamey J. M. (2015). Cold and hot extremozymes: industrial relevance and current trends. Front. Bioeng. Biotechnol. 3:148. 10.3389/fbioe.2015.00148 26539430 PMC4611823

[B153] Sathya T. A. Khan M. (2014). Diversity of glycosyl hydrolase enzymes from metagenome and their application in food industry. J. Food Sci. 79, R2149–R2156. 10.1111/1750-3841.12677 25311940

[B154] Schmidt T. M. Delong E. F. Pace N. R. (1991). Analysis of a marine picoplankton community by 16S rRNA gene cloning and sequencing. J. Bacteriol. 173, 4371–4378. 2066334 10.1128/jb.173.14.4371-4378.1991 PMC208098

[B155] Schoenfeld T. Patterson M. Richardson P. M. Wommack K. E. Young M. Mead D. (2008). Assembly of viral metagenomes from yellowstone hot springs. Appl. Environ. Microbiol. 74, 4164–4174. 10.1128/AEM.02598-07 18441115 PMC2446518

[B156] Schoenfeld T. W. Murugapiran S. K. Dodsworth J. A. Floyd S. Lodes M. Mead D. A.. (2013). Lateral gene transfer of family a DNA polymerases between thermophilic viruses, aquificae, and apicomplexa. Mol. Biol. Evol. 30, 1653–1664. 10.1093/molbev/mst078 23608703 PMC3684859

[B157] Schröder C. Elleuche S. Blank S. Antranikian G. (2014). Characterization of a heat-active archaeal β-glucosidase from a hydrothermal spring metagenome. Enzyme Microb. Technol. 57, 48–54. 10.1016/j.enzmictec.2014.01.010 24629267

[B158] Sen S. K. Jana A. Bandyopadhyay P. Das Mohapatra P. K. Raut S. (2016). Thermostable amylase production from hot spring isolate Exiguobacterium sp: a promising agent for natural detergents. Sustain. Chem. Pharm. 3, 59–68. 10.1016/j.scp.2016.04.002

[B159] Servín-Garcidueñas L. E. Peng X. Garrett R. A. Martínez-Romero E. (2013). Genome sequence of a novel archaeal rudivirus recovered from a mexican hot spring. Genome Announc. 1, e00040-12. 10.1128/genomeA.00040-12 23405288 PMC3569270

[B160] Shah N. Tang H. Doak T. G. Ye Y. (2011). Comparing bacterial communities inferred from 16S rRNA gene sequencing and shotgun metagenomics. Pac. Symp. Biocomput. 17, 165–176. 10.1142/9789814335058 21121044

[B161] Shao H. Xu L. Yan Y. (2013). Isolation and characterization of a thermostable esterase from a metagenomic library. J. Ind. Microbiol. Biotechnol. 40, 1211–1222. 10.1007/s10295-013-1317-z 23934105

[B162] Sharma A. Gilbert J. A. Lal R. (2016). (Meta)genomic insights into the pathogenome of *Cellulosimicrobium cellulans*. Sci. Rep. 6:25527. 10.1038/srep25527 27151933 PMC4858710

[B163] Sharma A. Jani K. Shouche Y. S. Pandey A. (2015). Microbial diversity of the Soldhar hot spring, India, assessed by analyzing 16S rRNA and protein-coding genes. Ann. Microbiol. 65, 1323–1332. 10.1007/s13213-014-0970-4

[B164] Sharma N. Tanksale H. Kapley A. Purohit H. J. (2012). Mining the metagenome of activated biomass of an industrial wastewater treatment plant by a novel method. Indian J. Microbiol. 52, 538–543. 10.1007/s12088-012-0263-1 24293707 PMC3516636

[B165] Sharpton T. J. (2014). An introduction to the analysis of shotgun metagenomic data. Front. Plant Sci. 5:209. 10.3389/fpls.2014.00209 24982662 PMC4059276

[B166] Shi H. Zhang Y. Li X. Huang Y. Wang L. Wang Y.. (2013). A novel highly thermostable xylanase stimulated by Ca^2+^ from Thermotoga thermarum: cloning, expression and characterization. Biotechnol. Biofuels 6:26. 10.1186/1754-6834-6-26 23418789 PMC3598563

[B167] Silva G. G. Z. Green K. T. Dutilh B. E. Edwards R. A. (2015). SUPER-FOCUS: a tool for agile functional analysis of shotgun metagenomic data. Bioinformatics 32, 354–361. 10.1093/bioinformatics/btv584 26454280 PMC4734042

[B168] Singh A. Subudhi E. (2016). Profiling of microbial community of Odisha hot spring based on metagenomic sequencing. Genomics Data 7, 187–188. 10.1016/j.gdata.2016.01.004 26981405 PMC4778635

[B169] Singh R. Chopra C. Kumar V. (2015). Purification and characterization of CHpro1, a thermotolerant, alkali-stable and oxidation-resisting protease of Chumathang hotspring. Sci. Bull. 60, 1252–1260. 10.1007/s11434-015-0834-8

[B170] Singh R. Dhawan S. Singh K. Kaur J. (2012). Cloning, expression and characterization of a metagenome derived thermoactive/thermostable pectinase. Mol. Biol. Rep. 39, 8353–8361. 10.1007/s11033-012-1685-x 22711301

[B171] Singhania R. R. Patel A. K. Sukumaran R. K. Larroche C. Pandey A. (2013). Role and significance of beta-glucosidases in the hydrolysis of cellulose for bioethanol production. Bioresour. Technol. 127, 500–507. 10.1016/j.biortech.2012.09.012 23069613

[B172] Sonbol S. A. Ferreira A. J. S. Siam R. (2016). Red Sea Atlantis II brine pool nitrilase with unique thermostability profile and heavy metal tolerance. BMC Biotechnol. 16:14. 10.1186/s12896-016-0244-2 26868129 PMC4751646

[B173] Song Z.-Q. Chen J.-Q. Jiang H.-C. Zhou E.-M. Tang S.-K. Zhi X.-Y.. (2010). Diversity of Crenarchaeota in terrestrial hot springs in Tengchong, China. Extremophiles 14, 287–296. 10.1007/s00792-010-0307-6 20373121

[B174] Song Z.-Q. Wang F.-P. Zhi X.-Y. Chen J.-Q. Zhou E.-M. Liang F.. (2013). Bacterial and archaeal diversities in Yunnan and Tibetan hot springs, China. Environ. Microbiol. 15, 1160–1175. 10.1111/1462-2920.12025 23126508

[B175] Stamps B. W. Corsetti F. A. Spear J. R. Stevenson B. S. (2014). Draft genome of a novel Chlorobi member assembled by tetranucleotide binning of a hot spring metagenome. Genome Announc. 2, e00897–e00814. 10.1128/genomeA.00897-14.Copyright 25212621 PMC4161750

[B176] Stetter K. O. (2006). Hyperthermophiles in the history of life. Philos. Trans. R. Soc. Lond. B Biol. Sci. 361, 1837–1842. 10.1098/rstb.2006.1907 17008222 PMC1664684

[B177] Suenaga H. Ohnuki T. Miyazaki K. (2007). Functional screening of a metagenomic library for genes involved in microbial degradation of aromatic compounds. Environ. Microbiol. 9, 2289–2297. 10.1111/j.1462-2920.2007.01342.x 17686025

[B178] Sun M. Z. Zheng H. C. Meng L. C. Sun J. S. Song H. Bao Y. J.. (2015). Direct cloning, expression of a thermostable xylanase gene from the metagenomic DNA of cow dung compost and enzymatic production of xylooligosaccharides from corncob. Biotechnol. Lett. 37, 1877–1886. 10.1007/s10529-015-1857-6 25994580

[B179] Sundarram A. Pandurangappa T. Murthy K. (2014). α -amylase production and applications : a review. J. Appl. Environ. Microbiol. 2, 166–175. 10.12691/jaem-2-4-10

[B180] Sundberg C. Al-Soud W. A. Larsson M. Alm E. Yekta S. S. Svensson B. H.. (2013). 454 pyrosequencing analyses of bacterial and archaeal richness in 21 full-scale biogas digesters. FEMS Microbiol. Ecol. 85, 612–626. 10.1111/1574-6941.12148 23678985

[B181] Tan H. Mooij M. J. Barret M. Hegarty P. M. Harington C. Dobson A. D. W.. (2014). Identification of novel phytase genes from an agricultural soil-derived metagenome. J. Microbiol. Biotechnol. 24, 113–118. 10.4014/jmb.1307.07007 24150499

[B182] Tan H. Wu X. Xie L. Huang Z. (2016). Identification and characterization of a mesophilic phytase highly resilient to high-temperatures from a fungus-garden associated metagenome. Appl. Microbiol. Biotechnol. 100, 2225–2241. 10.1007/s00253-015-7097-9 26536874

[B183] Tekere M. Lötter A. Olivier J. Jonker N. Venter S. (2011). Metagenomic analysis of bacterial diversity of Siloam hot water spring, Limpopo, South Africa. African J. Biotechnol. 10, 18005–18012. 10.5897/AJB11.899

[B184] Tsudome M. Deguchi S. Tsujii K. Ito S. Horikoshi K. (2009). Versatile solidified nanofibrous cellulose-containing media for growth of extremophiles. Appl. Environ. Microbiol. 75, 4616–4619. 10.1128/AEM.00519-09 19411423 PMC2704845

[B185] Uchiyama T. Miyazaki K. (2009). Functional metagenomics for enzyme discovery: challenges to efficient screening. Curr. Opin. Biotechnol. 20, 616–622. 10.1016/j.copbio.2009.09.010 19850467

[B186] Ufarté L. Potocki-Veronese G. Laville É. (2015). Discovery of new protein families and functions: new challenges in functional metagenomics for biotechnologies and microbial ecology. Front. Microbiol. 6:563. 10.3389/fmicb.2015.00563 26097471 PMC4456863

[B187] Urbieta M. S. Donati E. R. Chan K. G. Shahar S. Sin L. L. Goh K. M. (2015). Thermophiles in the genomic era: biodiversity, science, and applications. Biotechnol. Adv. 33, 633–647. 10.1016/j.biotechadv.2015.04.007 25911946

[B188] van Dijk E. L. Auger H. Jaszczyszyn Y. Thermes C. (2014). Ten years of next-generation sequencing technology. Trends Genet. 30, 418–426. 10.1016/j.tig.2014.07.001 25108476

[B189] de Vasconcellos S. P. Angolini C. F. F. García I. N. S. Martins Dellagnezze B. da Silva C. C. Marsaioli A. J. (2010). Screening for hydrocarbon biodegraders in a metagenomic clone library derived from Brazilian petroleum reservoirs. Org. Geochem. 41, 1067–1073. 10.1016/j.orggeochem.2010.08.003

[B190] Verma D. Kawarabayasi Y. Miyazaki K. Satyanarayana T. (2013). Cloning, expression and characteristics of a novel alkalistable and thermostable xylanase encoding gene (Mxyl) retrieved from compost-soil metagenome. PLoS ONE 8:e52459. 10.1371/journal.pone.0052459 23382818 PMC3561394

[B191] Verma D. Satyanarayana T. (2013). Improvement in thermostability of metagenomic GH11 endoxylanase (Mxyl) by site-directed mutagenesis and its applicability in paper pulp bleaching process. J. Ind. Microbiol. Biotechnol. 40, 1373–1381. 10.1007/s10295-013-1347-6 24100791

[B192] Vidal-Melgosa S. Pedersen H. L. Schückel J. Arnal G. Dumon C. Amby D. B.. (2015). A new versatile microarray-based method for high throughput screening of carbohydrate-active enzymes. J. Biol. Chem. 290, 9020–9036. 10.1074/jbc.M114.630673 25657012 PMC4423690

[B193] Vidya J. Swaroop S. Singh S. Alex D. Sukumaran R. Pandey A. (2011). Isolation and characterization of a novel α-amylase from a metagenomic library of Western Ghats of Kerala, India. Biologia 66, 939–944. 10.2478/s11756-011-0126-y

[B194] Wagner I. D. Wiegel J. (2008). Diversity of thermophilic anaerobes. Ann. N.Y. Acad. Sci. 1125, 1–43. 10.1196/annals.1419.029 18378585

[B195] Wang C. Dong D. Wang H. Müller K. Qin Y. Wang H.. (2016). Metagenomic analysis of microbial consortia enriched from compost: new insights into the role of Actinobacteria in lignocellulose decomposition. Biotechnol. Biofuels 9:22. 10.1186/s13068-016-0440-2 26834834 PMC4731972

[B196] Wang H. Gong Y. Xie W. Xiao W. Wang J. Zheng Y.. (2011). Identification and characterization of a novel thermostable gh-57 gene from metagenomic fosmid library of the juan de fuca ridge hydrothemal vent. Appl. Biochem. Biotechnol. 164, 1323–1338. 10.1007/s12010-011-9215-1 21455739

[B197] Wang M. Lai G. L. Nie Y. Geng S. Liu L. Zhu B.. (2015). Synergistic function of four novel thermostable glycoside hydrolases from a long-term enriched thermophilic methanogenic digester. Front. Microbiol. 6:509. 10.3389/fmicb.2015.00509 26052323 PMC4441150

[B198] Wang S. D. Guo G. S. Li L. Cao L. C. Tong L. Ren G. H.. (2014). Identification and characterization of an unusual glycosyltransferase-like enzyme with β-galactosidase activity from a soil metagenomic library. Enzyme Microb. Technol. 57, 26–35. 10.1016/j.enzmictec.2014.01.007 24629264

[B199] Wang S. Wang K. Li L. Liu Y. (2013). Isolation and characterization of a novel organic solvent-tolerant and halotolerant esterase from a soil metagenomic library. J. Mol. Catal. B Enzym. 95, 1–8. 10.1016/j.molcatb.2013.05.015

[B200] Wang Y. Qian P. Y. (2009). Conservative fragments in bacterial 16S rRNA genes and primer design for 16S ribosomal DNA amplicons in metagenomic studies. PLoS ONE 4:e7401. 10.1371/journal.pone.0007401 19816594 PMC2754607

[B201] Wemheuer B. Taube R. Akyol P. Wemheuer F. Daniel R. (2013). Microbial diversity and biochemical potential encoded by thermal spring metagenomes derived from the Kamchatka peninsula. Archaea 2013:136714. 10.1155/2013/136714 23533327 PMC3600328

[B202] Wierzbicka-Woś A. Bartasun P. Cieśliński H. Kur J. (2013). Cloning and characterization of a novel cold-active glycoside hydrolase family 1 enzyme with β-glucosidase, β-fucosidase and β-galactosidase activities. BMC Biotechnol. 13:22. 10.1186/1472-6750-13-22 23497058 PMC3605331

[B203] Wilson M. S. Siering P. L. White C. L. Hauser M. E. Bartles A. N. (2008). Novel archaea and bacteria dominate stable microbial communities in North America's largest hot spring. Microb. Ecol. 56, 292–305. 10.1007/s00248-007-9347-6 18080156

[B204] Wu M. Scott A. J. (2012). Phylogenomic analysis of bacterial and archaeal sequences with AMPHORA2. Bioinformatics 28, 1033–1034. 10.1093/bioinformatics/bts079 22332237

[B205] Xie W. Wang F. Guo L. Chen Z. Sievert S. M. Meng J.. (2011). Comparative metagenomics of microbial communities inhabiting deep-sea hydrothermal vent chimneys with contrasting chemistries. ISME J. 5, 414–426. 10.1038/ismej.2010.144 20927138 PMC3105715

[B206] Yap W. H. Zhang Z. Wang Y. (1999). Distinct types of rRNA operons exist in the genome of the actinomycete Thermomonospora chromogena and evidence for horizontal transfer of an entire rRNA operon. J. Bacteriol. 181, 5201–5209. 10464188 10.1128/jb.181.17.5201-5209.1999 PMC94023

[B207] Ye M. Li G. Liang W. Q. Liu Y. H. (2010). Molecular cloning and characterization of a novel metagenome-derived multicopper oxidase with alkaline laccase activity and highly soluble expression. Appl. Microbiol. Biotechnol. 87, 1023–1031. 10.1007/s00253-010-2507-5 20358193

[B208] Zamora M. A. Pinzón A. Zambrano M. M. Restrepo S. Broadbelt L. J. Moura M. (2015). A comparison between functional frequency and metabolic flows framed by biogeochemical cycles in metagenomes: the case of “El Coquito” hot spring located at Colombia's national Nevados park. Ecol. Modell. 313, 259–265. 10.1016/j.ecolmodel.2015.06.041

[B209] Zhang X. Li H. Li C.-J. Ma T. Li G. Liu Y.-H. (2013). Metagenomic approach for the isolation of a thermostable β-galactosidase with high tolerance of galactose and glucose from soil samples of Turpan Basin. BMC Microbiol. 13:237. 10.1186/1471-2180-13-237 24156692 PMC4016535

[B210] Zhou J. Bruns M. A. Tiedje J. M. (1996). DNA recovery from soils of diverse composition. Appl. Environ. Microbiol. 62, 316–322. 8593035 10.1128/aem.62.2.316-322.1996 PMC167800

[B211] Zhu Y. Li J. Cai H. Ni H. Xiao A. Hou L. (2013). Characterization of a new and thermostable esterase from a metagenomic library. Microbiol. Res. 168, 589–597. 10.1016/j.micres.2013.04.004 23684391

